# Influence of the Alternative Sigma Factor RpoN on Global Gene Expression and Carbon Catabolism in Enterococcus faecalis V583

**DOI:** 10.1128/mBio.00380-21

**Published:** 2021-05-18

**Authors:** Erica C. Keffeler, Vijayalakshmi S. Iyer, Srivatsan Parthasarathy, Matthew M. Ramsey, Matthew J. Gorman, Theresa L. Barke, Sriram Varahan, Sally Olson, Michael S. Gilmore, Zakria H. Abdullahi, Emmaleigh N. Hancock, Lynn E. Hancock

**Affiliations:** a Department of Molecular Biosciences, University of Kansas, Lawrence, Kansas, USA; b Division of Biology, Kansas State University, Manhattan, Kansas, USA; c Department of Ophthalmology, Harvard Microbial Sciences Initiative, Harvard Medical School, Massachusetts Eye and Ear Infirmary, Boston, Massachusetts, USA; d Comparative Medicine Group, Kansas State University, Manhattan, Kansas, USA; Nanyang Technological University

**Keywords:** CcpA, endocarditis, *Enterococcus faecalis*, RpoN, UTI, microarrays

## Abstract

The alternative sigma factor σ^54^ has been shown to regulate the expression of a wide array of virulence-associated genes, as well as central metabolism, in bacterial pathogens. In Gram-positive organisms, the σ^54^ is commonly associated with carbon metabolism. In this study, we show that the Enterococcus faecalis alternative sigma factor σ^54^ (RpoN) and its cognate enhancer binding protein MptR are essential for mannose utilization and are primary contributors to glucose uptake through the Mpt phosphotransferase system. To gain further insight into how RpoN contributes to global transcriptional changes, we performed microarray transcriptional analysis of strain V583 and an isogenic *rpoN* mutant grown in a chemically defined medium with glucose as the sole carbon source. Transcripts of 340 genes were differentially affected in the *rpoN* mutant; the predicted functions of these genes mainly related to nutrient acquisition. These differentially expressed genes included those with predicted catabolite-responsive element (*cre*) sites, consistent with loss of repression by the major carbon catabolite repressor CcpA. To determine if the inability to efficiently metabolize glucose/mannose affected infection outcome, we utilized two distinct infection models. We found that the *rpoN* mutant is significantly attenuated in both rabbit endocarditis and murine catheter-associated urinary tract infection (CAUTI). Here, we examined a *ccpA* mutant in the CAUTI model and showed that the absence of carbon catabolite control also significantly attenuates bacterial tissue burden in this model. Our data highlight the contribution of central carbon metabolism to growth of E. faecalis at various sites of infection.

## INTRODUCTION

Enterococci have emerged as leading causes of hospital-associated infections that are often associated with biofilms, including endocarditis and catheter-associated urinary tract infections (CAUTI) (1). Both disease manifestations for endocarditis and CAUTI are thought to be biofilm mediated, and enterococci now rank as the second leading cause of CAUTI in U.S. hospitals (1). The ability of enterococci to cause such infections is in part due to their ability to adapt and survive in a variety of host environments that are often nutrient poor (2). Critical nutrient substrates that permit microbial proliferation and induce pathology at the site of infection remain to be defined for many types of infection. While several studies have examined transcriptional profiles of enterococci grown in urine, serum, and abscesses (3–5), little is known of preferred nutrients and how E. faecalis acquires them in the host.

We previously showed that the alternative sigma factor σ^54^ (RpoN) contributes to *in vitro* biofilm formation in E. faecalis, as an *rpoN* deletion mutant was shown to have an altered biofilm matrix composition when grown in rich medium (6). The *rpoN* mutant was less efficient at autolysis (less extracellular DNA [eDNA] in the matrix), and the biofilm became more labile than the parental strain to protease K treatment. In addition, σ^54^ (RpoN) is also known to regulate several phosphotransferase systems (PTS) in E. faecalis, including a mannose/glucose permease, Mpt (7), making it a good candidate for exploring its contribution to *in vivo* fitness.

In contrast to other sigma factors, σ^54^ is unable to initiate open complex formation upon association with target DNA and the core RNA polymerase and requires the assistance of a bacterial enhancer binding protein (bEBP) (8). In E. faecalis, four bEBPs are encoded on the V583 genome (MptR, MpoR, MphR, and LpoR), with a fifth (XpoR) disrupted by an insertion element (7). The genes encoding each of these bEBPs are positioned immediately upstream of their respective sugar PTS genes (7). The best-characterized PTS in E. faecalis is the aforementioned Mpt mannose/glucose permease (7, 9, 10), owing to the fact that components of this PTS complex are known cellular receptors for class IIa and IIc bacteriocins (11).

Work by Opsata et al. (10) characterized the transcriptional profile of a mutant of *mptD*, a component of the Mpt PTS complex, that conferred resistance to pediocin PA-1, a known class IIa bacteriocin. These authors identified a number of differentially expressed genes in the *mptD* mutant that contained putative catabolite-responsive element (*cre*) sites based on similarity to consensus *cre* sites from Bacillus subtilis (12). *cre* sites are pseudopalindromes and are considered a low-conservation consensus sequence of WTGNNARCGNWWWCAW (strongly conserved residues are underlined) (13). In low-GC-content Gram-positive bacteria, *cre* sites on DNA are bound by a protein complex consisting of the carbon catabolite repressor CcpA and the phosphorylated Ser-46 form of Hpr (Hpr-46-P) (14). CcpA is a global regulatory protein that plays a critical role in regulating the expression of genes involved in secondary catabolite uptake and utilization in Gram-positive bacteria (15).

Because σ^54^ has been shown to regulate the expression of various genes involved in metabolism and virulence in other bacteria (16–20), we performed a microarray transcriptional analysis to identify genes whose expression was differentially expressed in the E. faecalis V583 strain and an isogenic Δ*rpoN* mutant. Furthermore, we also tested the role of RpoN and CcpA in biofilm formation under drip-flow conditions, as well as colonization *in vivo* using a rabbit endocarditis infection model and a murine model of catheter-associated urinary tract infection to determine whether RpoN-dependent metabolic pathways contribute to biofilm-associated infections. To our knowledge, this report represents the first examination of the contribution of either σ^54^ (RpoN) or CcpA to *in vivo* fitness in E. faecalis. Overall, this study provides important evidence linking basic metabolism with *in vivo* growth and provides the rationale for several distinct pathways that could be targeted as a potential therapeutic for treating enterococcal infections.

## RESULTS

### RpoN and MptR regulate glucose uptake and are essential for mannose utilization.

**FIG 1:**
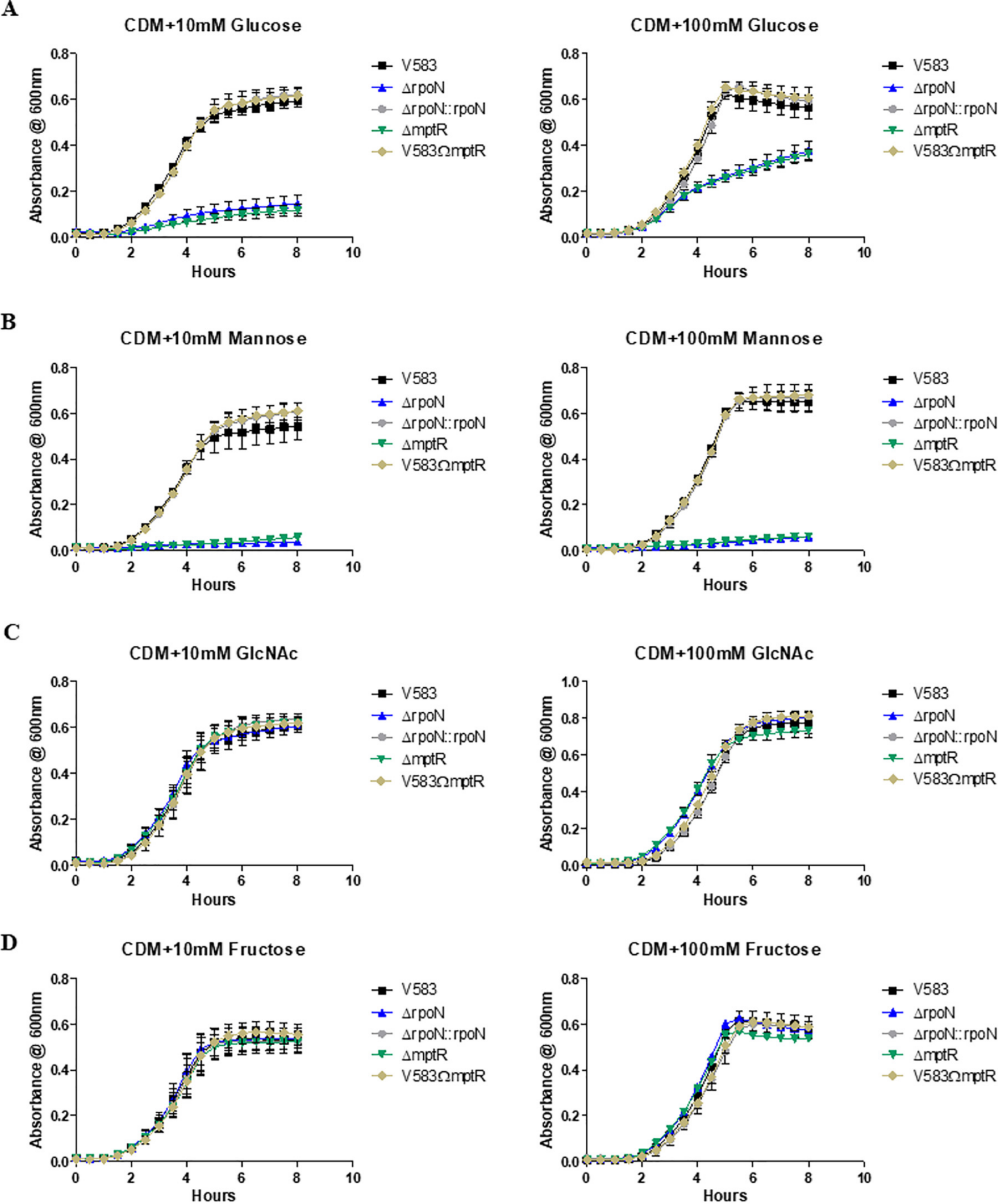
Growth of Enterococcus faecalis in chemically defined medium (CDM) with the sugars indicated as the principal carbon sources: (A) glucose, (B) mannose; (C) *N*-acetylglucosamine, and (D) fructose. The respective sugar concentrations are indicated above each graph. Each graph is the average of three biological replicates, with three technical replicates each time (*n* = 9) and standard error of the mean shown. The growth curves for each strain are shown in black (V583), blue (Δ*rpoN*), green (Δ*mptR*), gray (Δ*rpoN*::*rpoN*), and gold (V583Ω*mptR* revertant).

We previously demonstrated that deletion of *rpoN* has no growth defect in rich media, including tryptic soy broth and Todd-Hewitt broth (THB) (6). Several sugar uptake systems in E. faecalis are known to be regulated by RpoN (7). Therefore, it was of interest to determine whether RpoN contributed to fitness in a chemically defined medium (CDM) (21, 22) supplemented with various sugars as the sole carbon source. As RpoN also requires a bEBP for open complex formation in order for transcription to proceed, we also evaluated the contribution of the four bEBPs in E. faecalis V583 by constructing deletion mutants for *lpoR*, *mphR*, *mpoR*, and *mptR*. The fifth bEBP (XpoR) identified in E. faecalis possesses a natural insertion of an IS*256* element in the *xpoR* gene in the E. faecalis V583 genome. Because of the location of the IS*256* insertion within *xpoR*, it was unclear whether XpoR function was disrupted by the IS*256* element; we therefore constructed a deletion mutant that removed the corresponding *xpoABCD* PTS system and assessed its contribution directly. The deletion of *mphR*, *mpoR*, *lpoR*, or *xpoABCD* did not impact growth when glucose or mannose was present as the sole carbon source (see Fig. S1 in the supplemental material). In contrast, the *rpoN* and *mptR* deletion mutants displayed poor growth. Figure 1A shows that both the *rpoN* and *mptR* mutants grew very poorly in CDM supplemented with 10 mM glucose, and that this phenotype is complementable, as the *rpoN* complement and the *mptR* genetic revertant grew at a rate equivalent to that of the parental strain. Because deletion of *rpoN* and *mptR* had such a drastic impact on glucose-dependent growth, it was of interest to determine whether a homolog of the primary glucose transporter (PtsG) in Bacillus subtilis contributed to glucose uptake in E. faecalis. We therefore constructed a deletion mutant of *ef1516* and assessed its growth in CDM supplemented with either 10 mM or 100 mM glucose (see Fig. S2 in the supplemental material). EF1516 shares approximately 39% amino acid sequence identity and 56% sequence similarity with PtsG in B. subtilis. We did not detect a role for EF1516 in growth with glucose as the sole carbon source in E. faecalis, as there was no significant difference in growth between the *ef1516* mutant and the parental V583 strain (Fig. S2).

10.1128/mBio.00380-21.1 FIG S1 Growth of Enterococcus faecalis in CDM supplemented with 10 mM glucose or mannose. Each graph is the average of three biological replicates, with three technical replicates each time (*n* = 9) and standard error of the mean shown. The growth curves for each strain are represented as black (V583), blue (Δ*rpoN*), green (Δ*mptR*), red (Δ*mpoR*), yellow (Δ*mphR*), purple (Δ*lpoR*), pink (Δ*xpoABDC*), and orange (Δ*mptBACD*). Download FIG S1, DOCX file, 0.04 MB. Copyright © 2021 Keffeler et al. 2021 Keffeler et al. https://creativecommons.org/licenses/by/4.0/ This content is distributed under the terms of the Creative Commons Attribution 4.0 International license.

10.1128/mBio.00380-21.2 FIG S2 Growth of E. faecalis activator mutants in CDM containing 10 mM or 100 mM glucose. Each graph is the average of three internal replicates, repeated three times, with standard error of the mean shown. The growth curves for each strain are represented as black (V583), blue (Δ*rpoN*), green (Δ*mptR*), and orange (Δ*ef1516*). Download FIG S2, DOCX file, 0.03 MB. Copyright © 2021 Keffeler et al. 2021 Keffeler et al. https://creativecommons.org/licenses/by/4.0/ This content is distributed under the terms of the Creative Commons Attribution 4.0 International license.

To confirm that the poor growth of *mptR* was related to the direct regulation of the Mpt PTS system, we also constructed and evaluated an *mptBACD* mutant. The *mptBACD* mutant phenocopied the growth of *mptR*, suggesting that the inability of *rpoN* and *mptR* mutants to activate expression of the Mpt PTS system is responsible for their poor growth in glucose-dependent conditions (Fig. S1). At 100 mM glucose, the *rpoN* and *mptR* mutants showed improved growth but never achieved the maximal growth observed with the parental strain (optical density at 600 nm [OD_600_], ∼0.4 versus ∼0.6), suggesting alternative routes of glucose uptake at increasing concentrations of glucose. In contrast, the growth defect of the mutants in CDM that included mannose as the principal carbon source was not rescued by increasing the concentration of mannose (Fig. 1B), suggesting that the Mpt PTS controlled by σ^54^ and MptR represents the sole mannose transporter in the cell.

To ascertain the sugar specificity of the PTS controlled by MptR and σ^54^, we also grew cells in CDM with *N*-acetylglucosamine (GlcNAc) and fructose as a carbon source. As shown in Fig. 1C and D, the absence of either σ^54^ or MptR does not alter growth when *N*-acetylglucosamine or fructose is the main carbon source.

### Transcriptional analysis of the E. faecalis V583 Δ*rpoN* strain.

RpoN orthologs impact global gene expression in a variety of bacteria, but the genes that they regulate are functionally divergent (19, 23, 24). In E. faecalis, the only genes predicted to be directly regulated by RpoN are putative PTS operons, each of which contains the distinct −24/−12 promoter element (TTGGCACNNNNNTTGCT) thought to be responsive to RpoN (7). Because of its role in glucose uptake, we hypothesized that RpoN likely affects a larger regulatory gene network at the transcriptional level. Therefore, using DNA microarrays of E. faecalis strain V583, the transcriptional profile of the V583 Δ*rpoN* strain was compared to those of the parental strain V583 and an *rpoN*-complemented strain.

**FIG 2:**
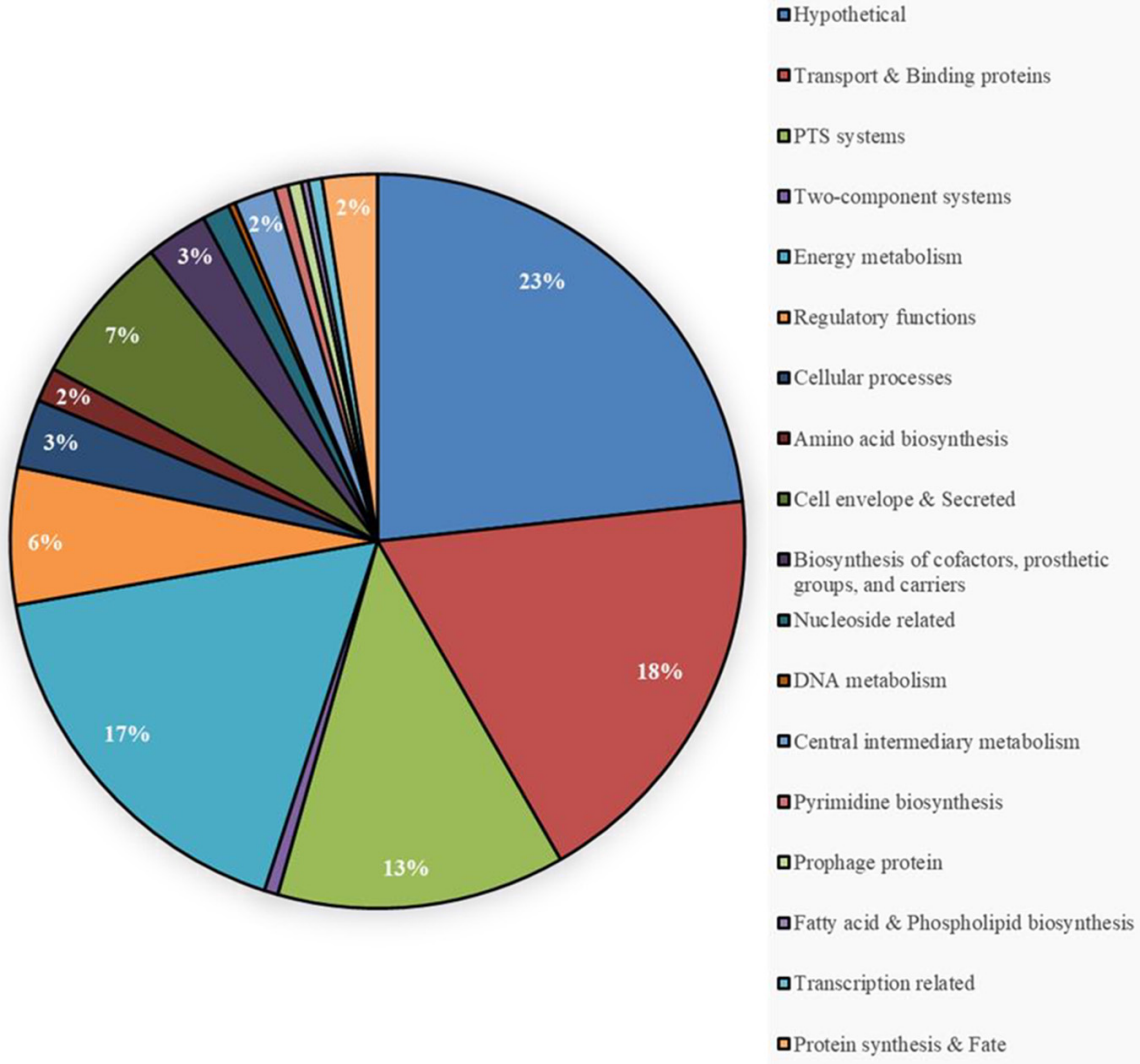
Pie chart depicting the functional distribution of the 340 differentially regulated genes in the Δ*rpoN* mutant. The number in each functional category is the percentage of the total differentially regulated genes. PTS, phosphotransferase system.

Compared to the parental strain and the *rpoN*-complemented strain, mRNA abundance in the *rpoN* mutant differed for transcripts corresponding to 340 genes (Fig. 2; see also Table S4 in the supplemental material). Of the 340 differentially expressed genes in the V583 Δ*rpoN* strain, transcripts for 255 genes were increased and those for 85 genes were decreased (≥3-fold) compared to those of the parental and complemented strains. Of the differentially regulated genes, 23% are predicted to encode hypothetical proteins with no known function, 18% encode transport and binding proteins, 17% are energy metabolism related, and 13% encode PTS proteins. Of the six PTS systems known to contain the −24/−12 RpoN binding site in their promoter element (Mpt, Mpo, Mph, Lpo, Lpt, and Xpo), three were significantly downregulated (*mpt* [*ef0019-22*], *xpo* [*ef3210-13*], and *lpt* [*ef1017-19*] by 152-fold, 6-fold, and 4-fold, respectively), and no significant difference in expression was observed in the *lpo*, *mpo*, and *mph* PTS systems.

10.1128/mBio.00380-21.7 TABLE S3 Plasmids used in this study. Download Table S3, DOCX file, 0.01 MB. Copyright © 2021 Keffeler et al. 2021 Keffeler et al. https://creativecommons.org/licenses/by/4.0/ This content is distributed under the terms of the Creative Commons Attribution 4.0 International license.

10.1128/mBio.00380-21.8 TABLE S4 Differentially expressed genes in the V583 Δ*rpoN* strain compared to the V583 strain. Download Table S4, DOCX file, 0.05 MB. Copyright © 2021 Keffeler et al. 2021 Keffeler et al. https://creativecommons.org/licenses/by/4.0/ This content is distributed under the terms of the Creative Commons Attribution 4.0 International license.

### Catabolite repression elements (*cre*) in differentially expressed genes in the V583 Δ*rpoN* strain.

The increased expression levels of genes encoding several sugar uptake systems, as well as ABC transporters, in the transcription profile of the *rpoN* mutant suggested a loss of catabolite control. In order to determine the basis of such regulation, we searched the regions upstream of the differentially expressed genes in the *rpoN* mutant to identify *cre* sites. We used the *cre* consensus sequences suggested for low-GC-content Gram-positive bacteria, 5′-WTGNNARCGNWWWCAW-3′ (13) and 5′-WTGWAARCGYWWWCW-3′ (12), as our pattern queries and used the Regulatory Sequence Analysis Tool (http://rsat.ulb.ac.be/), allowing for a 1-bp mismatch to identify *cre* sites upstream of the genes that were upregulated in the *rpoN* mutant. Of the 255 genes that were upregulated in the *rpoN* mutant, *cre* sites were present in the promoter regions of 46 genes; due to some loci comprising operons, this accounts for 109 genes, which represents 42.74% of the upregulated genes in the *rpoN* mutant (see Table S5 in the supplemental material). It is of note that we identified a three-gene operon (*ef1017-19*), which encodes the Lpt PTS complex that was downregulated in the *rpoN* mutant that also contained a *cre* site, indicating that this operon may also be regulated by CcpA for expression.

10.1128/mBio.00380-21.9 TABLE S5 Putative *cre* sites in differentially expressed genes in the V583 Δ*rpoN* strain compared to the V583 strain. Download Table S5, DOCX file, 0.04 MB. Copyright © 2021 Keffeler et al. 2021 Keffeler et al. https://creativecommons.org/licenses/by/4.0/ This content is distributed under the terms of the Creative Commons Attribution 4.0 International license.

### Identification of cell envelope-associated or secreted gene products among the differentially expressed genes in the *rpoN* mutant that contain *cre* sites.

To identify cell envelope-associated or secreted gene products from the pool of differentially expressed genes in the *rpoN* mutant that also contained *cre* sites, we analyzed the 112 differentially regulated gene products (109 upregulated and 3 downregulated in an *rpoN* mutant) identified in Table S5 for the presence of a signal peptide sequence and signal peptidase cleavage site (SignalP 5.0) followed by analysis for the presence of transmembrane domains (TMHMM/TMpred) or LPXTG cell wall-anchoring motifs using PHI-BLAST and the pattern query for the sortase cell wall-sorting signal (L-P-[SKTAQEHLDN]-[TA]-[GN]-[EDASTV]) (25) with the E. faecalis collagen adhesion protein, Ace, as the template query (26). Identification of predicted lipoproteins was performed using Pred-Lipo. As summarized in Table 1, 36 of the 112 gene products analyzed contained a signal peptide sequence and/or predicted transmembrane helices (TMHs), suggesting that these differentially expressed and *cre* site-containing genes encode proteins that are cell envelope-associated or secreted. Among these 36 genes, 27 encode predicted transport proteins, five of which (*ef1234*, *ef1397*, *ef2221*, *ef2234*, and *ef2237*) encode predicted lipoproteins involved in substrate binding associated with ABC transporters. One notable PTS system potentially regulated by the presence of a predicted *cre* site near the translation start site was EF0551-55. This PTS system resides within the known pathogenicity island present in V583 (27). In addition to transport functions, a putative family 8 polysaccharide lyase (EF3023) and a family 31 glycosyl hydrolase (EF1824) were the only gene products from this cohort that contain a predicted LPXTG cell wall-anchoring motif, suggesting that these proteins are anchored on the bacterial cell surface and interact with the external environment. Last, three of the 31 genes listed in Table 1 (*ef0114*, *ef0361*, and *ef2863*) encode glycosyl hydrolases thought to be secreted into the external environment, as they contain predicted signal peptides and signal peptidase cleavage sites. Encoded in the same operon as *ef0361*, *ef0362* encodes a chitin binding protein that is also predicted to be secreted into the surrounding environment. Overall, this set of *in silico* analyses indicates that a relatively small cohort of differentially expressed genes in the *rpoN* mutant that also contain predicted *cre* sites are likely to be involved in the interaction of the bacterial cell with its environment.

**TABLE 1:** Putative cell-enveloped associated or secreted gene products that are differentially regulated in the *rpoN* mutant and also possess *cre* sites

Operon	Gene	Function	Fold change	Start	End	*cre* sequence of operon or gene^*a*^	Signal peptide probability^*c*^	No. of TMHs^*d*^
EF0104-08	EF0108	C4-dicarboxylate transporter; putative	13.04	−146	−132	ATGAAAGCGCATTCT	0.997	13
	EF0114	Glycosyl hydrolase, family 20	48.20	−35	−21	GTGTATGCGCTTTCT	0.980	1
EF0292-91	EF0292	PTS system, IIC component	6.62	−51	−37	ATGTAAACGGATACA	NA	10
EF0362-61	EF0361	Chitinase, family 2	69.31	−41	−27	CTGTAAGCGCATACA	1.000	0
	EF0362	Chitin binding protein; putative	83.75	−41	−27	CTGTAAGCGCATACA	1.000	1
	EF0439	Immunity protein PlnM; putative	13.54	−49	−35	ATGAAAACGTTATCA	NA	2
EF0551-55	EF0552	PTS system, IIC component	6.21	−85	−70	ATACAAACGCTTTCAT	NA	6
	EF0553	PTS system, IID component	11.35	−85	−70	ATACAAACGCTTTCAT	NA	5
	EF0569	Potassium-transporting ATPase, subunit C	−3.16	−169	−154	ATGCTAGTGGAATCAA	0.979	1
	EF0635	Amino acid permease family protein	−5.11	−270	−255	TTAGGAGCGTAAACAT	0.920	12
EF1017-19	EF1019	PTS system, IIB component	−4.49	−173	−158	TTGGAAACGCACACAA	NA	8
	EF1207	Citrate carrier protein, CCS family	8.54	−53	−39	ATGTAAACGTTTTCT	NA	12
EF1232-34	EF1232	ABC transporter, permease protein	8.63	−81	−67	ATGTAAGGGTTTACA	NA	6
	EF1233	ABC transporter, permease protein	13.83	−81	−67	ATGTAAGGGTTTACA	0.999	6
	EF1234	ABC transporter, substrate binding protein; putative	12.52	−81	−67	ATGTAAGGGTTTACA	1.000	6
EF1392-1400	EF1397	Molybdenum ABC transporter, molybdenum binding protein	23.85	−38	−24	GTGTAAACGTTAACA	0.999	1
	EF1398	Molybdenum ABC transporter, permease protein	26.30	−38	−24	GTGTAAACGTTAACA	NA	5
	EF1400	Cadmium-translocating P-type ATPase	6.43	−38	−24	GTGTAAACGTTAACA	NA	6
EF1663-1557	EF1657	PTS IIC membrane protein; putative	5.80	−61	−47	ATGTAAACGCATACA	0.765	8
	EF1800	Hypothetical protein	4.01	−61	−47	ATGAAAGCGTGTTCA	1.000	2
EF1805-01	EF1802	PTS system, IID component	7.35	−30	−16	TTGAAAGCGTTTACT	NA	5
	EF1803	PTS system, IIC component	8.28	−30	−16	TTGAAAGCGTTTACT	NA	7
	EF1824	Glycosyl hydrolase, family 31	6.82	−255	−241	ATGAAAACGCATTCA	0.827	1
EF1929-27^*b*^	EF1927	Glycerol uptake facilitator protein	36.40	−146	−132	TTGAAAGCGTTGTCT	0.724	6
				−36	−22	TTGAAATCGTTTTCT		
EF2223-21	EF2221	ABC transporter, substrate binding protein	128.09	−38	−24	ATGAAAACGCTATTA	1.000	1
	EF2222	ABC transporter, permease protein	114.80	−38	−24	ATGAAAACGCTATTA	0.996	6
	EF2223	ABC transporter, permease protein	257.25	−38	−24	ATGAAAACGCTATTA	0.986	6
EF2237-32	EF2232	ABC transporter, permease protein	4.20	−35	−21	ATTAAAGCGCTTTCT	0.997	6
	EF2233	ABC transporter, permease protein	8.46	−35	−21	ATTAAAGCGCTTTCT	0.972	5
	EF2234	Sugar ABC transporter, sugar binding protein; putative	17.24	−35	−21	ATTAAAGCGCTTTCT	1.000	1
	EF2237	Lipoprotein	7.17	−35	−21	ATTAAAGCGCTTTCT	1.000	1
	EF2863	Endo-beta-*N*-acetylglucosaminidase	62.34	−45	−31	TTGTAAGCGCTAACA	1.000	1
	EF3023	Polysaccharide lyase, family 8	8.85	−209	−195	GTGAAAGCGTAAACA	1.000	2
EF3142-34^*b*^	EF3138	PTS system, IID component	17.41	−374	−360	ATGAAAAGGCATTCA	NA	5
				−68	−34	ATGTAAACGATTACA		
	EF3139	PTS system, IIC component	7.55	−374	−360	ATGAAAAGGCATTCA	NA	7
				−68	−34	ATGTAAACGATTACA		
	EF3327	Citrate transporter	7.22	−45	−31	TTGTAAGCGTTAACA	NA	12

a Strongly conserved residues of the *cre* consensus sequence are underlined.

b Genes/operons with more than one predicted *cre* site.

c NA, not applicable.

d TMH, transmembrane helix.

### Quantitative real-time PCR of differentially expressed genes in the *rpoN* mutant that are CcpA dependent and independent.

**FIG 3:**
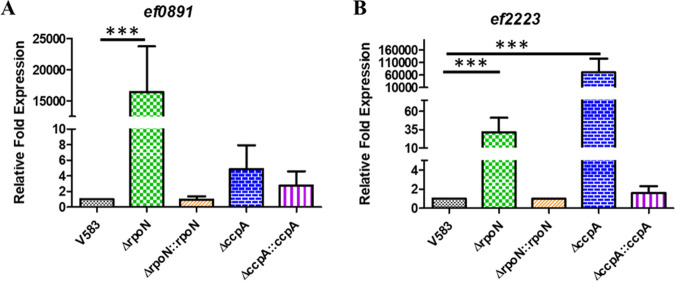
Quantitative real-time PCR (qRT-PCR) analysis of selected CcpA-dependent and independent regulated genes that were highly upregulated in Δ*rpoN*. RNA was isolated from mid-log-phase cultures of V583, Δ*rpoN*, Δ*rpoN*::*rpoN*, Δ*ccpA*, and Δ*ccpA*::*ccpA* strains grown in chemically defined medium supplemented with 100 mM glucose; RNA was subsequently converted to cDNA. The cDNA was subjected to qPCR analysis and quantified using the threshold cycle (ΔΔ*C_T_*) method with the threshold cycle values for the gene of interest, namely *ef0891* (A) and *ef2223* (B, normalized to the endogenous control (*ef0005* [*gyrB*]). Results represent the averages of three independent biological experiments. Error bars indicate the standard deviation of the mean. Statistical analysis was done by one-way analysis of variance (ANOVA), with significance values set to *P* < 0.0001 (***).

To validate the microarray data, we performed quantitative real-time PCR (qRT-PCR) on a set of genes that represented both upregulated (*ef0891* and *ef2223*) and downregulated (*ef0019* [*mptB*], *ef1017* [*lptB*], and *ef3210* [*xpoA*]) genes in the *rpoN* mutant. This list of validated genes included those whose expression profiles were found to be in common with the previous *mptR* and *mptD* mutant transcriptomes (*ef0019* and *ef2223*) (10), as well as those genes that were found to be unique to the *rpoN* mutant (*ef1017*, *ef3210*, and *ef0891*). We examined the expression of *ef0891* and *ef2223* in the *rpoN* mutant by using qRT-PCR to compare transcript abundance to that in the parental and *rpoN*-complemented strains. The results shown in Fig. 3A and B validate the array data, as relative expression for *ef0891* and *ef2223* was significantly increased in the *rpoN* mutant. The expression of *ef2223-21*, an operon encoding an ABC transporter, was the most abundantly upregulated transcript in the *rpoN* mutant that also contained a *cre* site within the promoter region (Tables S4 and S5). To confirm whether the expression of *ef2223* is also CcpA dependent, qRT-PCR was performed on RNA isolated from the wild-type, *ccpA* mutant, and *ccpA*-complemented strains. Figure 3B shows that the expression of *ef2223* is highly upregulated in the *ccpA* mutant background relative to the parental V583 strain and *ccpA* complement. In contrast, *ef0891* expression appears to be independent of CcpA, as there was no significant difference in *ef0891* expression in the *ccpA* mutant background relative to the parental V583 and complemented strains (Fig. 3A). This result indicates that the expression of *ef2223* is likely indirectly upregulated as a consequence of *rpoN* deletion and is directly regulated by CcpA due to the presence of a predicted *cre* site near its promoter region. In contrast, the differential expression of *ef0891* is unique to the *rpoN* mutant, as no change was observed in a *ccpA* mutant.

**FIG 4:**
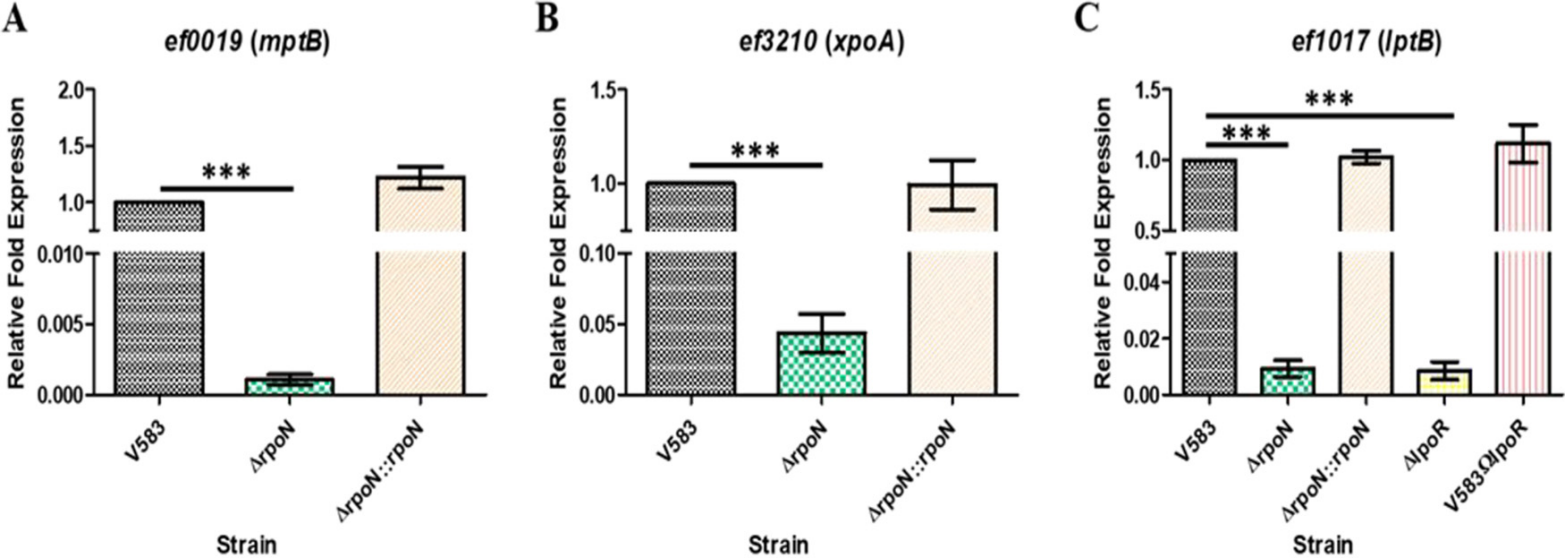
qRT-PCR analysis of RpoN-dependent expression of (A) *ef0019* (*mptB*) and (B) *ef3210* (*xpoA*). RNA was isolated from cultures of V583, Δ*rpoN*, and Δ*rpoN*::*rpoN* strains grown in CDM supplemented with 100 mM glucose and was subsequently converted to cDNA. (C) qRT-PCR analysis of RpoN- and LpoR-dependent expression of *ef1017* (*lptB*). RNA was isolated from cultures of V583, Δ*rpoN*, Δ*rpoN*::*rpoN*, Δ*lpoR*, and V583Ω*lpoR* revertant strains grown in CDM supplemented with 100 mM glucose and was subsequently converted to cDNA. The cDNA was subjected to qPCR analysis and quantified using the ΔΔ*C_T_* method with the threshold cycle values for *ef0019*, *ef1017*, and *ef3210* normalized to the endogenous control (*ef0005* [*gyrB*]). Results represent averages of three independent biological experiments. Error bars indicate the standard deviation of the mean. Statistical analysis was done by one-way ANOVA, with significant values set to *P* < 0.0001 (***).

To confirm expression of genes that were downregulated in the *rpoN* mutant, we focused on three PTS operons predicted to contain the consensus −24/−12 RpoN-dependent promoter (*ef0019*, *ef1017*, and *ef3210).* The results shown in Fig. 4A to C confirmed that these three genes were significantly downregulated in the *rpoN* mutant relative to the parental V583 and *rpoN*-complemented strains.

The most downregulated genes in the *rpoN* mutant array were the *mptBACD* operon encoding the Mpt PTS system; this was confirmed in the qRT-PCR data using *mptB* (*ef0019*), expression of which was reduced ∼930-fold in the *rpoN* mutant compared to that in the parental strain. In addition to *mpt* genes, we also confirmed reduced expression of the *lptBAC* operon using *lptB* (*ef1017*), which was reduced ∼100-fold in the *rpoN* mutant, and the *xpoABCD* operon using *xpoA* (*ef3210*), which was reduced ∼20-fold (Fig. 4). These observations are consistent with RpoN playing a direct role in the regulation of these PTS systems, as the promoters for each of these PTS systems contains a predicted −24/−12 consensus RpoN-dependent promoter. As the *lptBAC* operon is unique among RpoN-dependent genes in that it is not immediately proceeded by a gene encoding a bEBP, we hypothesized that LpoR (*ef1010*) is likely involved in regulating the expression of the *lptBAC* (*ef1017-19*) PTS operon, as it is encoded in close proximity. We observed by qRT-PCR that the deletion of *lpoR* resulted in an ∼100-fold decrease in expression of *lptB* (*ef1017*) in CDM supplemented with 100 mM glucose relative to that in the parental and *lpoR* genetic revertant strains (Fig. 4C). This suggests that the LpoR bEBP is involved in activating the expression of the *lpt* PTS operon.

**FIG 5:**
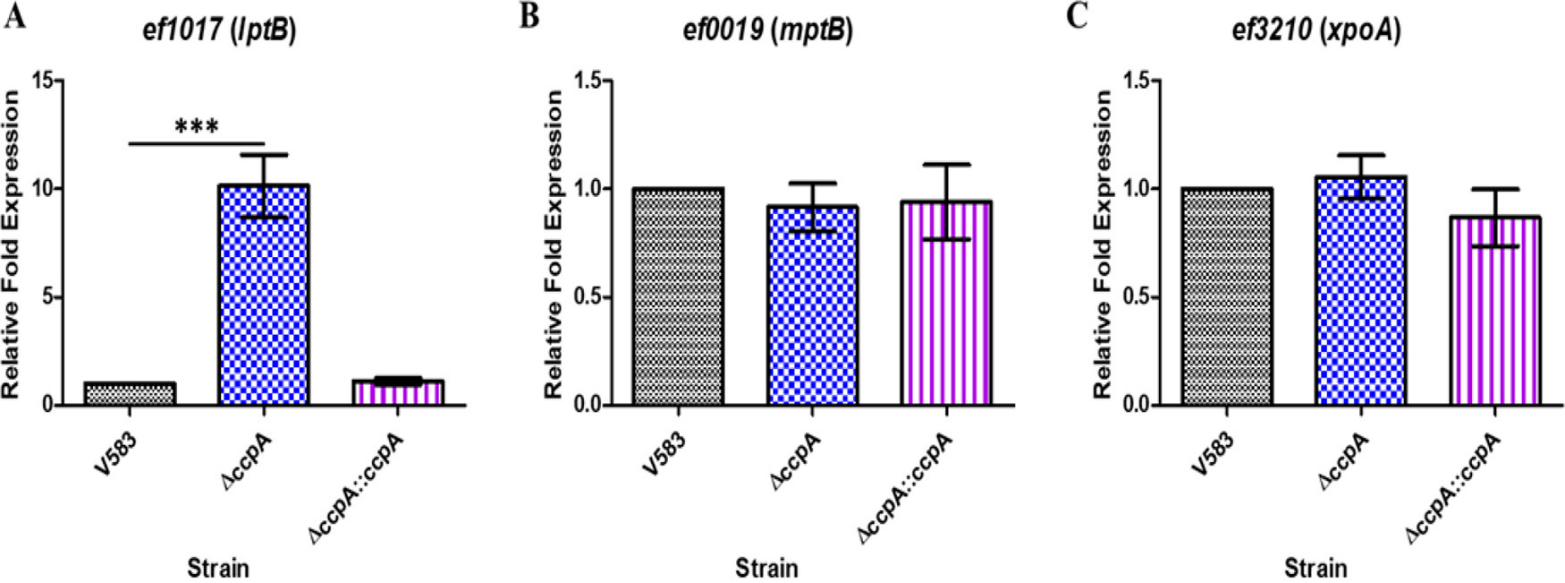
qRT-PCR analysis of selected CcpA-dependent and independent regulated genes that are RpoN dependent. RNA was isolated from mid-log cultures of V583, Δ*ccpA*, and Δ*ccpA*::*ccpA* strains grown in CDM supplemented with 100 mM glucose and was subsequently converted to cDNA. The cDNA was subjected to qPCR analysis and quantified using the ΔΔ*C_T_* method with the threshold cycle values for the gene of interest, namely *ef1017* (A), *ef0019* (B), and *ef3210* (C), normalized to the endogenous control (*ef0005* [*gyrB*]). Results represent the averages of three independent biological experiments. Error bars indicate the standard deviation of the mean. Statistical analysis was done by one-way ANOVA, with significance values set to *P* < 0.0001 (***).

Of the differentially expressed genes that were downregulated in the *rpoN* mutant, only the *lptBAC* promoter region possessed a putative *cre* site (Table S5). To confirm whether the regulation of the *lpt* PTS operon is also dependent on CcpA, qRT-PCR was performed on RNA isolated from wild-type, *ccpA* mutant, and *ccpA*-complemented strains grown to the mid-exponential phase in CDM supplemented with 100 mM glucose. Figure 5A shows that the expression of *lptB* (*ef1017*) is upregulated 10-fold in the *ccpA* mutant background relative to that in the parental V583 and *ccpA*-complemented strains. In contrast, *mptB* (*ef0019*) and *xpoA* (*ef3210*) expression appears to be independent of CcpA, as there was no significant difference in expression in the *ccpA* mutant background relative to that in the parental V583 and complemented strains (Fig. 5B and C).

### The EF2223-21 ABC transporter contributes to glucose importation.

**FIG 6:**
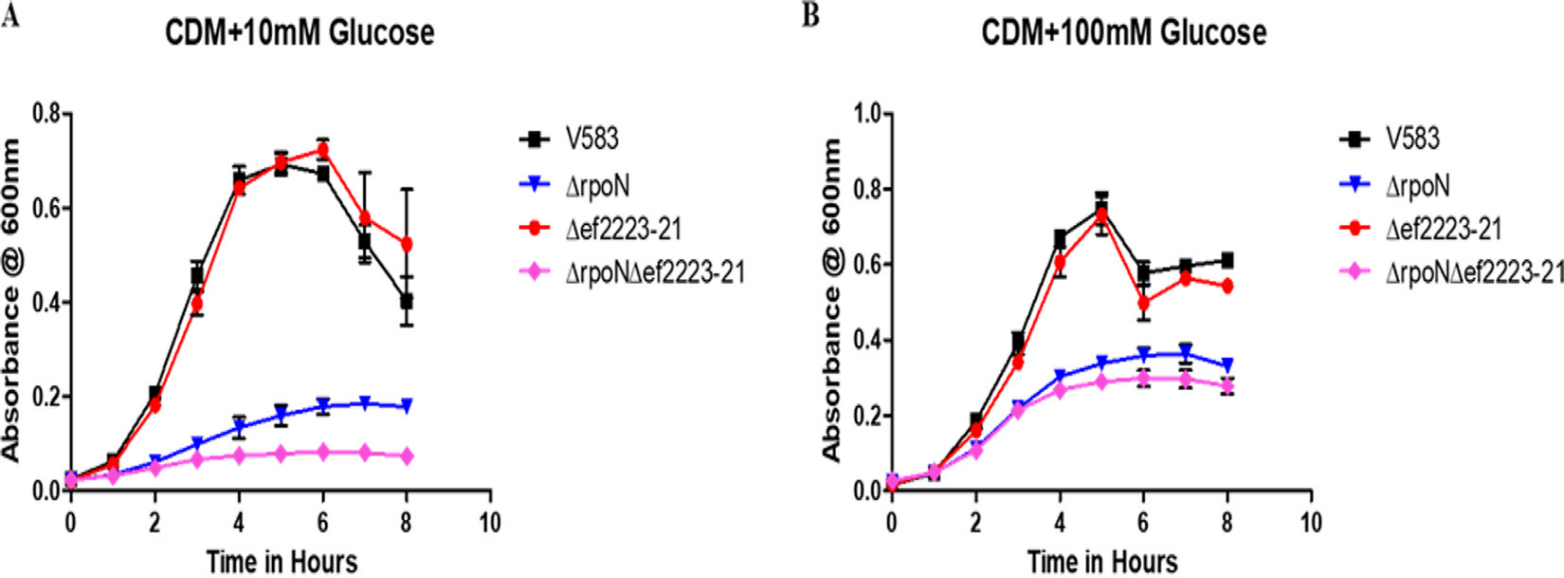
Growth of E. faecalis in chemically defined medium with glucose as the sole carbon source. The respective glucose concentrations are indicated below each panel. Each graph is the average of three biological replicates, with three technical replicates each time (*n* = 9) and standard error of the mean shown. The growth curves for each strain are shown in black (V583), blue (Δ*rpoN*), red (Δ*ef2223–ef2221*), and pink (Δ*rpoN* Δ*ef2223–ef2221*).

In the presence of high glucose concentrations (100 mM), the growth defect in both the *mptR* and *rpoN* mutants appeared to be partially rescued, suggesting that at higher concentrations, glucose is being transported via other uptake system(s). The upregulation of several PTS systems and transport proteins in the *rpoN* mutant gives credence to this hypothesis. Of note is gene *ef2223*, which encodes an ABC transporter permease protein, a member of the *ef2223-21* ABC transporter operon that was found to be 257.25-fold upregulated in the *rpoN* mutant, possesses a *cre* site, and was also confirmed to be highly upregulated in the *ccpA* mutant (Table S4 and Fig. 3). We hypothesized that when the Mpt PTS system is incapable of importing glucose efficiently into the cell as a consequence of the *rpoN* deletion, CcpA will derepress to allow expression of *ef2223-21* in order to bring glucose into the cell as a non-PTS glucose importer. To test this hypothesis, *ef2223-21* deletion mutants, singly and in combination with the Δ*rpoN* mutant, were grown in CDM supplemented with 10 mM or 100 mM glucose. Figure 6 illustrates that a single deletion of *ef2223-21* does not impede the overall growth relative to that of the wild type in CDM supplemented with either concentration of glucose. However, a double deletion mutant of *rpoN* and *ef2223-21* results in a significant attenuation in growth relative to that of V583 Δ*rpoN* alone; this reduction in growth is most pronounced in CDM supplemented with 10 mM glucose. At the higher concentration of glucose (100 mM), the difference in growth of the double mutant (Δ*rpoN* Δ*ef2223–ef2221*) relative to that of the *rpoN* mutant is less pronounced, suggesting that additional low-affinity glucose transporters likely contribute to glucose importation.

### Role of RpoN and CcpA in enterococcal biofilm formation.

**FIG 7:**
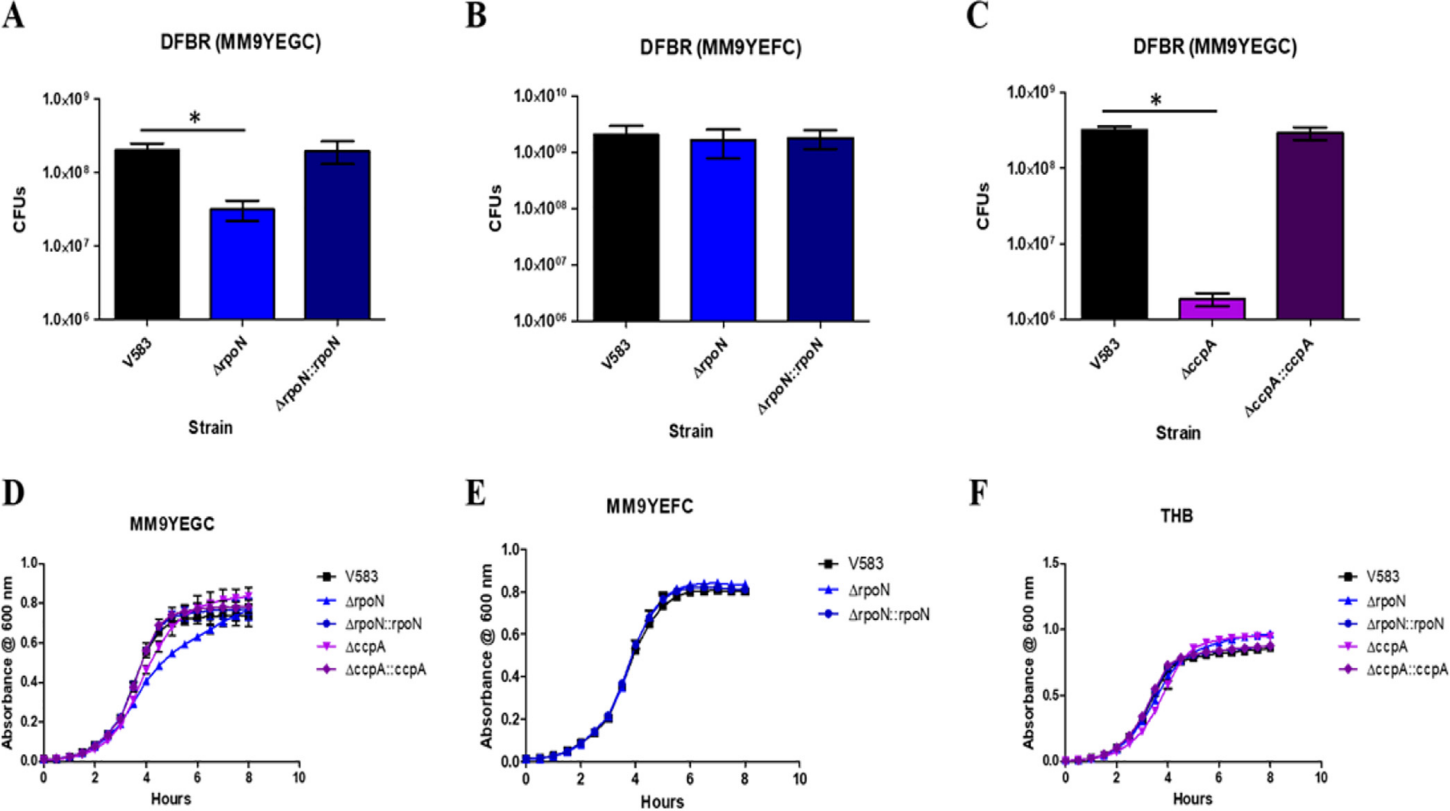
Quantification of biofilm formation of V583, Δ*rpoN*, and Δ*rpoN*::*rpoN* strains using a drip-flow biofilm reactor (DFBR) in (A) MM9YEGC and (B) MM9YEFC media. (C) Quantification of biofilm formation of V583, Δ*ccpA*, and Δ*ccpA*::*ccpA* strains using a drip-flow biofilm reactor (DFBR) in MM9YEGC medium. Results represent the averages of three independent biological experiments with error bars indicating the standard deviation of the mean. Statistical analysis was done by one-way ANOVA, with significance values set to *P* < 0.05 (*). (D to F) Growth of E. faecalis in (D) MM9YEGC, (E) MM9YEFC, and (F) THB media. Each graph is the average of three biological replicates, with three technical replicates each time (*n* = 9) and standard error of the mean shown. The growth curves for each strain are shown in black (V583), blue (Δ*rpoN*), dark blue (Δ*rpoN*::*rpoN*), purple (Δ*ccpA*), and dark purple (Δ*ccpA*::*ccpA*).

We have previously shown that the E. faecalis V583 Δ*rpoN* strain exhibited resistance to autolysis and formed altered biofilm structures in which the matrix was more protease K labile (6). The biofilm conditions in the prior study were based on a static biofilm under nutrient-rich conditions, and we were interested in knowing how RpoN and CcpA might contribute to biofilm formation under flow conditions under nutrient-poor conditions to more closely mimic the environment that is likely encountered at sites of infection. To assess the role of RpoN and CcpA in regulating biofilm formation, we quantified the level of biofilm formation under flow conditions of *rpoN* and *ccpA* mutant strains using a drip-flow bioreactor (DFBR) (28, 29) in 0.1× MM9YEGC medium (modified M9 medium supplemented with yeast extract, glucose, and casamino acids). In the absence of *rpoN*, there is a significant 6-fold decrease in biofilm formation relative to that of the parental V583 strain and *rpoN* complement, whereas in the absence of *ccpA*, there is a more drastic decrease in biofilm formation that is represented by an approximate 170-fold decrease in biofilm formation compared to that of parental V583 or the *ccpA*-complemented strain (Fig. 7A and C). This suggests that factors regulated by RpoN and CcpA play a role in enterococcal biofilm formation. The *rpoN* mutant did exhibit a slight planktonic growth defect in the biofilm growth medium (MM9YEGC) (Fig. 7D), whereas there was no significant difference in growth relative to that of V583 in rich laboratory medium (Todd-Hewitt broth) (Fig. 7F). In contrast to the *rpoN* mutant, the *ccpA* mutant did not exhibit significant differences in planktonic growth relative to that of the parental V583 strain in biofilm growth medium (MM9YEGC) or in rich laboratory medium (Todd-Hewitt broth) (Fig. 7D and F), indicating that the biofilm growth defect observed in the *ccpA* mutant is unique to the biofilm microenvironment. To ascertain whether the biofilm phenotype associated with the *rpoN* mutant was linked to the inability to import the available carbon source in the biofilm medium, the *rpoN* mutant was assessed under drip-flow biofilm conditions with the biofilm growth medium containing fructose instead of glucose. Under these biofilm growth conditions, there was no significant difference between the *rpoN* mutant and the wild-type and the *rpoN*-complemented strains (Fig. 7B).

### Role of RpoN and CcpA in enterococcal virulence.

**FIG 8:**
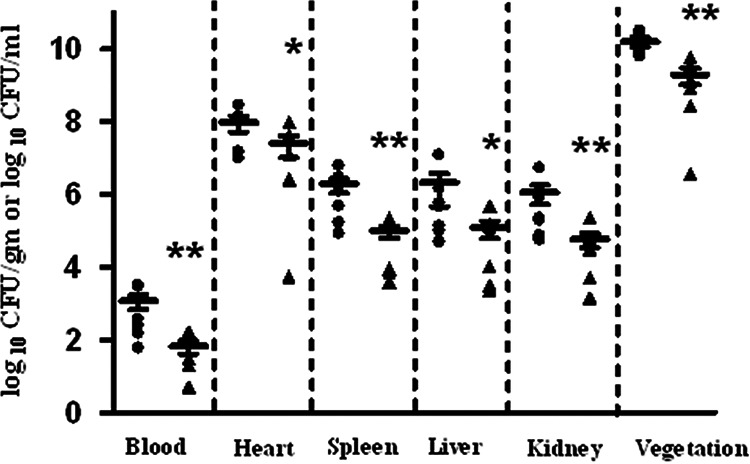
Enterococcal burden in rabbits infected with E. faecalis strains. Rabbits were euthanized postinfection, and organs were harvested to enumerate bacterial burden. Bacterial burden for wild-type V583 (●) and Δ*rpoN* (▴) are expressed as log_10_ CFU/g of harvested tissue. The horizontal line represents the median value for each group. A Mann-Whitney test was used to determine significance, which is indicated as follows: **, significant *P* value < 0.05 relative to V583; *, significant *P* value < 0.1 relative to V583.

**FIG 9:**
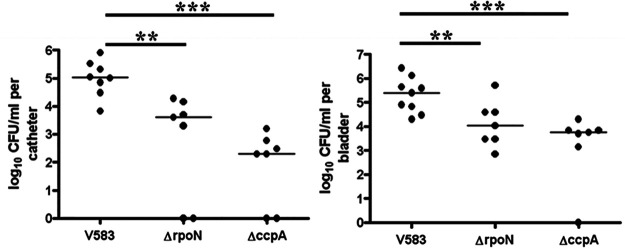
RpoN and CcpA contribute to enterococcal virulence in the murine model of CAUTI. Female C57BL/6 mice were euthanized after 48 h postinfection. Bacterial burden is expressed in a logarithmic scale for wild-type V583, Δ*rpoN*, and Δ*ccpA* strains in (A) implanted catheters retrieved from the mice and (B) homogenized bladders. The horizontal bar represents the median of each group of mice. Statistical significance as determined by Mann-Whitney test is represented as follows: **, significant *P* value < 0.05 relative to V583; ***, significant *P* value < 0.005 relative to V583.

On the basis of the biofilm results and the observation that RpoN plays a key role in regulating the uptake of mannose and glucose, we hypothesized an important contribution of RpoN to the *in vivo* fitness of E. faecalis. To determine the role of enterococcal RpoN in virulence we used two models of infection, rabbit endocarditis (30) and a murine model of catheter-associated urinary tract infection (31). In the rabbit endocarditis model, the parental strain (V583) was compared to its isogenic *rpoN* mutant for the ability to establish infective endocarditis, and mean bacterial burden on the heart valve vegetation, heart, liver, spleen, and kidneys, as well as in the blood, were assessed. As observed in Fig. 8, a significant reduction (*P* < 0.05) of approximately 10-fold in the mean bacterial burden in comparison to that of the parental strain was noted in the blood and in all examined organs of the rabbits infected with Δ*rpoN*, suggesting that *rpoN* contributes to infective endocarditis in rabbits. We also assessed the contributions of both RpoN and CcpA in the murine model of CAUTI. Similarly to the observation in the endocarditis model, the *rpoN* mutant was significantly attenuated in CAUTI, as shown by the reduced numbers of bacteria (*P* < 0.05) recovered from the bladder (6.7-fold lower) and catheter (32.6-fold lower) of the Δ*rpoN* strain-infected mice compared to those infected with the wild-type strain V583 (Fig. 9). In comparison to the wild-type, the *ccpA* mutant was more highly attenuated for *in vivo* fitness, as the mean bacterial numbers for the *ccpA* mutant isolated from the catheter and bladder were 500-fold and 90-fold lower, respectively, than those of the wild type (*P* < 0.005).

## DISCUSSION

The sigma factor σ^54^ (RpoN) has historically been linked to the regulation of nitrogen metabolism, even before the protein was recognized as a sigma factor (32). However, it is now well established that RpoN regulates a plethora of cellular processes, including flagellar biosynthesis in Escherichia coli (23), cold shock adaptation in Bacillus subtilis (33), sporulation and toxin production in Bacillus cereus (34), biofilm formation in E. faecalis (6), and PTS-mediated regulation in Gram-negative and Gram-positive organisms (24, 35, 36). Others have conducted several comparative studies for understanding σ^54^ and bEBP-mediated regulation with the intent of identifying a unifying biological theme for the wide range of RpoN-dependent processes (37–39). Francke et al. performed an extensive comparative genomic analyses and proposed that σ^54^ is a central player in the control of cellular processes that involve the physical interaction of an organism with its environment (host colonization, biofilm, etc.) by directly regulating the expression of genes involved in the transport and biosynthesis of the main precursors of the bacterial exterior (40).

σ^54^ binding sites (TTGGCACNNNNNTTGCT) have previously been identified upstream of genes predicted to encode sugar PTS systems (Mpt, Mpo, Lpo, Lpt, Mph, and Xpo) in E. faecalis, and each PTS system is thought to be regulated by a bEBP that is predicted to interact with RpoN to allow open complex formation (7). Of the six predicted RpoN-dependent PTS systems, three (*mpt*, *lpt*, and *xpo*) were found to be differentially expressed under CDM plus glucose growth conditions, but only the Mpt system plays a significant role in glucose- and mannose-dependent growth, as the *mptR* mutant and the *mptBACD* mutant phenocopy the *rpoN* mutant for growth in CDM supplemented with glucose or mannose. We have also previously shown that an Δ*rpoN* mutant is resistant to 2-deoxyglucose (2DG; a toxic homologue of glucose) (6), and mutants resistant to 2DG have been shown to localize mutations within *mptR* or the *mptBACD* operon (7, 41) thus strengthening the notion that Mpt is the major glucose uptake system in E. faecalis.

In contrast, neither the other known bEBPs (MphR, MpoR, and LpoR) nor the XpoABCD PTS system contribute to glucose-dependent growth. We also show here for the first time that the *lptBAC* PTS operon expression is dependent on the LpoR bEBP. Why the Lpt and Xpo PTS systems are induced with glucose as a principal carbon source is unclear, as neither appears to contribute to glucose-dependent growth in a chemically defined medium. Previously, it was hypothesized that the Xpo PTS complex was inactive in E. faecalis V583 due to a truncated XpoR that lacks its C-terminal regulatory domain due to an IS*256* insertion in the *xpoR* gene (7). However, our results indicate that the expression of the *xpo* PTS system is dependent on RpoN, suggesting that the truncated XpoR may still be functional. Others have shown that a bEBP that only possesses its AAA+ ATPase domain is capable of stimulating transcription of RpoN-dependent promoters in Salmonella enterica subsp. *enterica* serovar Typhimurium LT2 (42), suggesting that, in some instances, bEBPs may activate transcription of its RpoN-dependent genes without possessing regulatory and/or DNA binding domains. Another possibility is that one of the other five bEBPs encoded in the E. faecalis V583 genome may be used to activate the RpoN-dependent *xpo* operon. Deciphering the sugar specificity of the Xpo system remains an active area of investigation.

One of the common themes observed with the transcriptional profile of the *rpoN* mutant was the significant upregulation of genes with predicted *cre* sites, suggestive of an involvement with the major catabolite control protein CcpA. Among the cohort of *cre*-regulated genes, the only *cre*-containing genes that were significantly downregulated in the *rpoN* microarray were those of the *lpt* PTS operon. Our observations confirm a role for CcpA in the regulation of the *lpt* PTS. In general, *cre* sites present in the promoter regions of genes repressed by CcpA overlap the –10 promoter element or can be found immediately downstream of the –10 box (43). Based on our *cre* site query, we identified a *cre* site located 158 bp upstream of the start codon of *ef1017* (*lptB*) and positioned 92 bp upstream of the predicted –24/–12 promoter. RpoN-mediated transcriptional activation requires the binding of bEBP to an upstream activation sequence (UAS) to facilitate open complex formation. The UAS is generally positioned 80 to 150 bp upstream of the –24/–12 promoter, and bEBP binding to the UAS requires DNA looping to bring the bEBP into close contact with the RNA polymerase (44). The location of the *cre* site positioned 92 bp upstream of the *lpt* operon –24/–12 promoter suggests that CcpA may actively compete with LpoR for binding to an as-yet-unidentified UAS. To our knowledge, this would be the first instance in which CcpA exhibits repressor activity by competing with a bEBP rather than with RNA polymerase to prevent transcription of target genes. Understanding the specifics of this observation, as well as the sugar specificity of the Lpt PTS complex remains a component of ongoing studies.

Based on the *in silico* analysis conducted in this study, among the cohort of *cre*-regulated genes, nearly a third (32.1%) are predicted to be cell envelope-associated or secreted gene products. The majority of the genes listed in Table 1 encode transport and transport binding proteins, including a novel PTS system (EF0551-55) that resides within a known pathogenicity island in strain V583 (27), potentially linking sugar metabolism with pathogenesis or increased competitive fitness in a complex intestinal ecology. The remaining *cre* site-regulated genes encode either cell wall-anchored proteins or secreted glycosyl hydrolases (exo- or endoglycosidases). Endoglycosidases are enzymes that function to release oligosaccharides from glycoproteins or glycolipids and do not require the presence of a terminal sugar residue to affect cleavage, thus distinguishing them from known exoglycosidases. The endoglycosidases also serve to release available nutrients from the host and can therefore be thought of as nutrient acquisition systems. The genes for each of the endoglycosidases (*ef0114* [*EndoE*]; *ef0362*-*ef0361* [*chiBA*], and *ef2863*) were ≥50-fold upregulated in the *rpoN* deletion mutant compared to V583 (see Tables S4 and S5 in the supplemental material). These genes have also been shown to be upregulated in various transcriptomic studies conducted in human urine, serum, and an *in vivo* subdermal abscess model (3–5), indicating biological host colonization relevance in an environment that is glucose limited. The contribution of these glycosyl hydrolases to E. faecalis virulence is a component of ongoing studies. With respect to the cohort of genes described in Table 1, we propose that the differentially expressed genes in the *rpoN* mutant that are putatively regulated by CcpA are involved in the physical interaction with its glucose-limited host environment, further supporting the findings in Francke et al. (40) that RpoN is involved in the central control of the bacterial exterior.

Leboeuf et al. showed that a *ccpA* insertion mutant in strain JH2-2 had a significantly altered growth rate in semisynthetic medium (Folic AOAC medium; Bacto) plus 0.15% glucose. A *ccpA* deletion mutant displayed a slightly altered growth in CDM with 10 mM glucose (see Fig. S3 in the supplemental material), but, in contrast, growth was not significantly altered in either THB or MM9YEGC. These combined observations suggest that growth alterations caused by *ccpA* gene disruption are likely to be dependent on growth medium as well as on strain. As previously stated, CcpA is known to play a critical role in secondary carbon metabolism by repressing secondary catabolite genes when preferred carbon sources are readily available, but CcpA also participates in the positive regulation of gene products known to be involved in central glycolytic pathways and overflow metabolism (45). Although secondary nutrient acquisition systems would be predicted to be overexpressed in a *ccpA* mutant, in our study this mutant performs poorly *in vivo*, as well as when grown under biofilm conditions *in vitro*. The dysregulation of normal central metabolism that would occur in a *ccpA* mutant likely explains its attenuated biofilm and *in vivo* phenotypes and is consistent with a growing body of evidence for the role of CcpA in Gram-positive bacterial pathogenesis (46–48). Of note, one of the E. faecalis lactate dehydrogenase genes, *ef0255* (*ldh-1*), contains a putative *cre* site. The location of the putative *cre* site within the promoter region of *ldh-1* predicts that CcpA acts as an activator of *ldh-1* expression. Leboeuf et al. (49) demonstrated by Northern blotting that *ldh-1* expression was marginally reduced (2-fold) in a *ccpA* insertion mutant, suggesting that CcpA partially contributes to the positive regulation of *ldh-1* expression. While we did not observe differential regulation of *ldh-1* expression in the *rpoN* mutant, Opsata et al. (10) observed a slight drop (2.4-fold) in expression of *ldh-1* in an *mptD* insertion mutant. Experimental differences in terms of strain background, medium composition, and growth phase between our study in comparison to other studies (10, 49) likely explain why we were unable to observe a decrease in *ldh-1* expression in our *rpoN* transcriptomic data.

10.1128/mBio.00380-21.3 FIG S3 Growth of E. faecalis in chemically defined medium (CDM) supplemented with 10 mM glucose. Each graph is the average of three biological replicates, with three technical replicates each time (*n* = 9) and standard error of the mean shown. The growth curves for each strain are shown in black (V583) and purple (Δ*ccpA*). Download FIG S3, DOCX file, 0.02 MB. Copyright © 2021 Keffeler et al. 2021 Keffeler et al. https://creativecommons.org/licenses/by/4.0/ This content is distributed under the terms of the Creative Commons Attribution 4.0 International license.

Ldh-1 has been linked to promoting extracellular electron transfer (EET) for biofilm matrix-associated iron-augmented energy production, thus leading to enhanced biofilm growth in E. faecalis (50). With this link between lactate dehydrogenase and biofilm formation, we hypothesize that when glucose is available, CcpA positively regulates the expression of *ldh-1*, thus leading to enhanced biofilm growth. In support of this hypothesis, increased glucose concentrations in biofilm culture medium have been shown to enhance biofilm formation in E. faecalis (51, 52), suggesting that replete glucose conditions during biofilm formation maintains CcpA-regulated *ldh* expression, thus leading to enhanced biofilm growth. We observed by qRT-PCR that a *ccpA* deletion mutant showed reduced *ldh-1* expression in CDM supplemented with 15 mM glucose (see Fig. S4 in the supplemental material). The inability to fully activate *ldh-1* expression in the *ccpA* mutant could partially explain the attenuated biofilm formation phenotypes we observed. Additionally, recent work by Kaval et al. (53) showed that CcpA regulates bacterial microcompartment (BMC) formation required for the utilization of ethanolamine in E. faecalis by binding to a *cre* site in the *eutS* promoter. The absence of CcpA predictably increases the expression of *eut* genes and BMC formation. The dysregulation of *eut* gene expression observed in a *ccpA* mutant likely results in increased BMC formation and a predictable decrease in cell growth, likely due to the metabolic demand placed on a cell to produce additional BMCs or the potential toxicity associated with ethanolamine utilization (54). This defect would be expected to be most pronounced in environments where ethanolamine utilization would occur, particularly in the gastrointestinal (GI) tract or possibly in other host anatomic sites, likely contributing to the *in vivo* defect of a *ccpA* mutant. Our *in vitro* growth conditions lacked supplemental ethanolamine, and we therefore did not observe a significant change in gene expression for *eut* genes. Collectively, the observations with the *rpoN* and *ccpA* mutants both *in vitro* and *in vivo* suggest that regulated metabolism is key to successful colonization and infection. Too little nutrient acquisition of essential host sugars (glucose and/or mannose) in the case of the *rpoN* mutant and dysregulated metabolism observed in the *ccpA* mutant result in poor fitness compared to that of the parental strain.

10.1128/mBio.00380-21.4 FIG S4 Quantitative real-time PCR (qRT-PCR) analysis of CcpA-dependent expression of *ef0255* (*ldh-1*). RNA was isolated from cultures of V583, Δ*ccpA*, and Δ*ccpA*::*ccpA* strains grown in CDM supplemented with 15 mM glucose and was subsequently converted to cDNA. The cDNA was subjected to qPCR analysis and quantified using the threshold cycle (ΔΔ*C_T_*) method, using the threshold cycle values for *ef0255* normalized to the endogenous control (*ef0005* [*gyrB*]). Results represent averages of three independent biological experiments. Error bars indicate the standard deviation of the mean. Statistical analysis was done by one-way analysis of variance (ANOVA), with significance set at *P* < 0.05 (*). Download FIG S4, DOCX file, 0.06 MB. Copyright © 2021 Keffeler et al. 2021 Keffeler et al. https://creativecommons.org/licenses/by/4.0/ This content is distributed under the terms of the Creative Commons Attribution 4.0 International license.

*In vivo*, the organism will face more hostile growth conditions, as preferable nutrient sources are kept at growth-limiting conditions (i.e., glucose is present in normal human serum at 4 to 8 mM [55] and similar blood glucose levels are also observed in rabbits [56]). Importantly, secondary carbon sources in a host environment could be host glycoproteins, such as high-mannose-type glycoproteins, which have been shown to serve as a potential nutrient source for E. faecalis *in vitro* (57). Thus, beyond glucose, an *rpoN* mutant’s failure to grow on mannose likely also contributes to its attenuated phenotype *in vivo*.

It was noteworthy that the most abundantly upregulated transcript in the *rpoN* mutant was a predicted sugar ABC transporter (EF2223-21). Deletion of this operon in V583 did not display a significant change in growth; however, deletion of *ef2223-21* in the *rpoN* mutant background resulted in further attenuation of growth in CDM with 10 mM glucose compared to the that of the *rpoN* mutant alone. Increasing the glucose concentration to 100 mM rescued this growth defect, suggesting that additional glucose transporters are present in E. faecalis. In both Staphylococcus aureus (58) and Streptococcus pyogenes (59), GlcU has been shown to contribute to glucose uptake under low affinity conditions, and a GlcU homolog is also present in the V583 genome (*ef0928*). Recent work by Kumar et al. (60) showed that GlcU expression compensates for PTS-dependent glucose transport when E. faecalis is exposed to the lantibiotic nisin. Their findings indicated that glucose was shuttled through the pentose phosphate shunt pathway under GlcU-dependent conditions, as opposed to the conventional glycolytic pathway. GlcU was first characterized in Bacillus subtilis by Paulsen et al. as a glucose:H^+^ symporter, which is dependent on the proton motive force for activity (61). It will be of interest to examine whether GlcU in E. faecalis is responsible for the improved growth of the *rpoN* and *ef2223-21* mutant under elevated glucose conditions. Although the nearest PtsG homolog in E. faecalis (EF1516) is not the primary glucose transporter when the RpoN-dependent Mpt PTS system is functional, we cannot rule out a glucose uptake contribution in the absence of the Mpt system. Intriguingly, the deletion of *rpoN* resulted in a 14-fold increase in *ef1516* expression, suggesting that it may play a role in glucose uptake, but this will require additional investigation. The gene encoding EF1516 does not appear to possess a *cre* site, so understanding how the absence of *rpoN* influences the expression of *ef1516* remains to be elucidated in subsequent studies.

**FIG 10:**
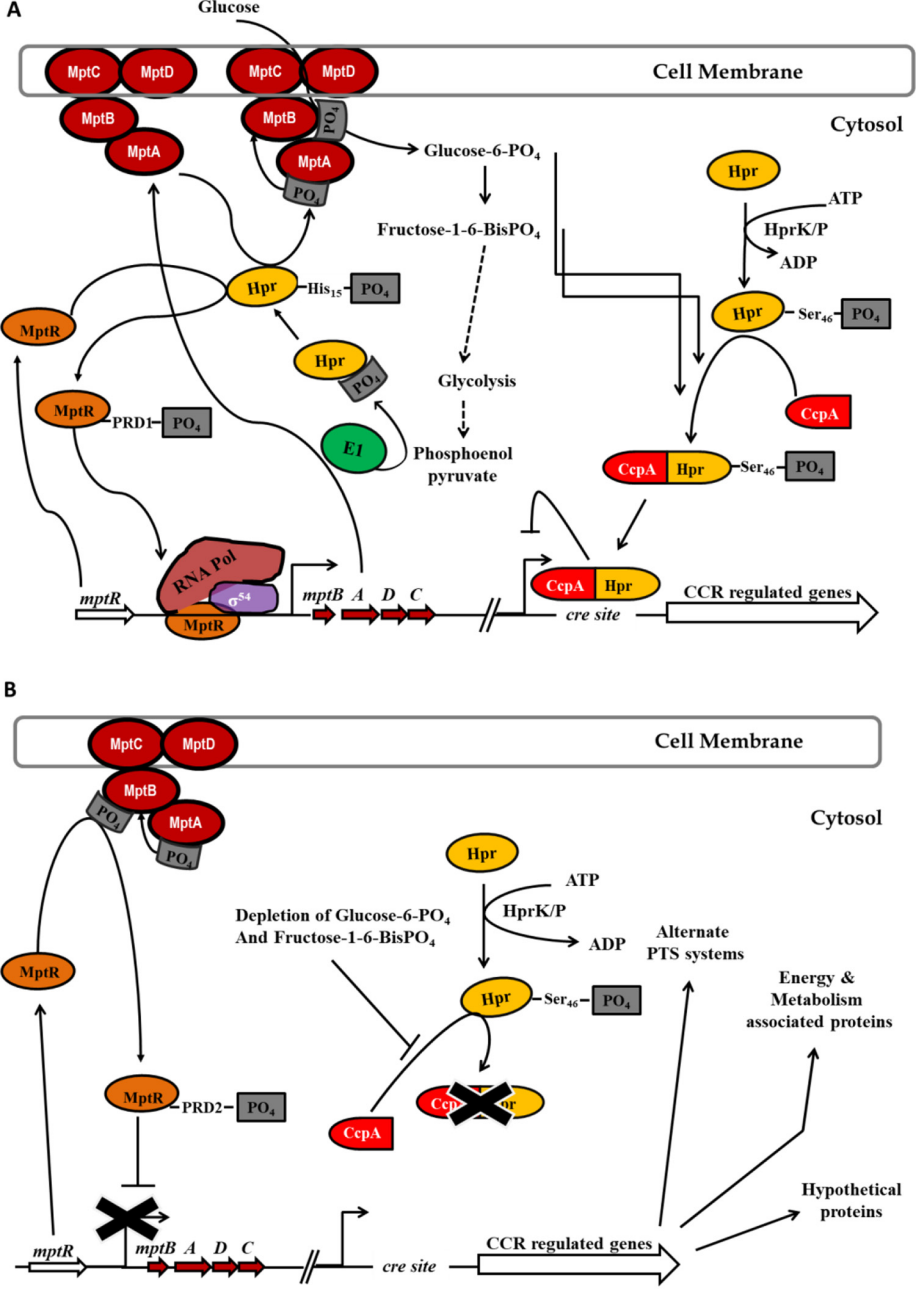
Model for σ^54^-mediated carbon catabolite repression (CCR). (A) Regulation of CCR-dependent genes in wild-type E. faecalis V583. (B) Alleviation of CCR in an *rpoN* or *mptR* mutant. CcpA, catabolite control protein A; Mpt, mannose PTS; *cre*, catabolite-responsive element; Hpr, histidine-containing phosphocarrier protein; EI, enzyme I; HprK/P, bifunctional ATP-dependent Hpr kinase/phosphatase; PRD, phosphotransferase regulation domain.

We present here a model for how RpoN and CcpA interface in the cell to regulate central carbon metabolism (Fig. 10). In the absence of RpoN, there would be an alteration in the relative abundance of the glycolytic intermediates glucose-6-phosphate and fructose-1,6-bisphosphate within the cell, indicating insufficient carbon flow, which would trigger the phosphatase activity of the bifunctional enzyme HprK/P that dephosphorylates the PTS intermediate Hpr(Ser-P) (Fig. 10). A dephosphorylated Hpr no longer binds to the catabolite control protein A (CcpA), and the dissociation of the Hpr(Ser-P)-CcpA complex would alleviate the repression of transcription of *cre*-dependent genes (20). In the Δ*rpoN* mutant, the inability to efficiently import glucose or mannose into the cell via the Mpt PTS complex influences the rate of carbon catabolite derepression. The expression seen in the array data with respect to *cre* site containing genes is consistent with this interpretation.

MptR belongs to the LevR-like family of regulators, whose bEBP activation is triggered by signal sensing through phosphotransferase regulation domains (PRDs) (8, 62). The activity of LevR-like regulators is controlled via phosphorylation of the bEBP regulatory domain by the PTS enzymes, which the bEBP regulates in turn. The regulatory domains of LevR-like bEBPs contain two unique PRDs; one domain undergoes HPr-mediated phosphorylation that leads to the activation of the bEBPs (PRD1), while PRD2 undergoes EII-mediated phosphorylation that is inhibitory (8, 62). It is of note that it has been described that when the substrate is present, EIIB preferentially phosphorylates the sugar, not the bEBP, to complete the PTS cascade (8, 62, 63). In the model depicted in fig. 10A, we also propose that when glucose is readily available, HPr becomes phosphorylated by EI via the phosphotransfer from the phosphoenolpyruvate (PEP) donor. HPr will transfer its phosphoryl group to the EII complex of the PTS system in addition to MptR PRD1. Phosphorylated MptR (MptR-PRD1) can then elicit open complex formation of RpoN to turn on expression of the *mpt* PTS complex. In contrast, when glucose is not readily available, MptR is phosphorylated by EIIB (MptR-PRD2), which inhibits MptR activation, thus inhibiting the expression of the *mpt* PTS genes (Fig. 10B).

In E. faecalis, the transcription profile of the *rpoN* mutant clearly shows that this sigma factor contributes to the activation of several PTS systems, as well as its impact on controlling the activity state of the Hpr (Ser-46) CcpA repressor. Approximately 10% of the genome is differentially expressed by disruption of RpoN function, and understanding these complex metabolic circuits that also likely feed into the virulence potential of E. faecalis will be the focus of ongoing studies.

## MATERIALS AND METHODS

### Bacterial strains and growth conditions.

Bacterial strains used in this study are listed in Table S1 in the supplemental material. For propagation of plasmids, Escherichia coli ElectroTen-Blue from Stratagene was cultivated in Luria-Bertani (LB) broth supplemented with appropriate antibiotics whenever necessary. Unless otherwise mentioned, E. faecalis was cultured in Todd-Hewitt broth (THB; BD Biosciences) containing appropriate antibiotics. For antibiotic selection, chloramphenicol (Cm) at a concentration of 10 μg/ml and 15 μg/ml was used for E. coli and E. faecalis, respectively.

10.1128/mBio.00380-21.5 TABLE S1 Enterococcus faecalis strains used in this study. Download Table S1, DOCX file, 0.01 MB. Copyright © 2021 Keffeler et al. 2021 Keffeler et al. https://creativecommons.org/licenses/by/4.0/ This content is distributed under the terms of the Creative Commons Attribution 4.0 International license.

### Construction of in-frame markerless deletion.

Using the temperature-sensitive cloning vector pLT06 (64), isogenic in-frame deletion mutants of the genes encoding the four bEBPs were generated in E. faecalis V583. Upstream and downstream flaking regions of the targeted activators were amplified using primers listed in Table S2 in the supplemental material. The primer pairs MptRP1/MptRP2 and MptRP3/MptRP4 were used to amplify flanking regions upstream and downstream of *mptR*, respectively. To facilitate cloning, primers MptRP1/MptRP2 were designed with EcoRI/BamHI restriction sites, respectively, whereas MptRP3/MptRP4 were designed with BamHI/PstI sites, respectively. For the construction of the insert, the amplified regions were digested with BamHI, ligated, and reamplified with MptRP1 and MptRP4. To generate pMG07 (*mptR* deletion vector), the amplified insert fragment was digested and ligated with EcoRI/PstI cut pLT06 cloning vector. The ligated vector and insert were electroporated into E. coli ElectroTen-Blue, and correct constructs were identified by colony PCR. The construct was screened by restriction digest analysis and then electroporated into competent E. faecalis V583 cells. The MG07 (V583Δ*mptR*) strain was subsequently generated as previously described (64) and confirmed by PCR using the primers MptR-Up and MptR-Down, and the paired genetic revertant from this screen was designated the MG07R (V583Ω*mptR*) strain. A similar approach was used to create all the remaining mutants and/or revertants used in this study (Table S1).

10.1128/mBio.00380-21.6 TABLE S2 Oligonucleotides used in this study. Download Table S2, DOCX file, 0.02 MB. Copyright © 2021 Keffeler et al. 2021 Keffeler et al. https://creativecommons.org/licenses/by/4.0/ This content is distributed under the terms of the Creative Commons Attribution 4.0 International license.

### Construction of in-frame markerless *ccpA*-complemented strain.

Using the temperature-sensitive cloning vector pLT06 (64), an isogeneic in-frame *ccpA*-complemented strain was generated in E. faecalis V583. The entirety of the *ccpA* gene, including its upstream and downstream flanking DNA regions, was amplified using the primer pair CcpAP1/CcpAP4 (Table S2). For cloning purposes, CcpAP1 and CcpAP4 were designed with EcoRI and PstI restriction sites, respectively. The amplified region was digested with EcoRI and PstI, ligated into the EcoRI/PstI-digested pLT06 cloning vector, and then electroporated into E. coli ElectroTen-Blue cells. The presence of the correct clone containing the recombinant plasmid was identified by colony PCR. The plasmid construct was confirmed by restriction digest analysis and sequenced. This plasmid was designated pEK26 and was subsequently electroporated into E. faecalis V583Δ*ccpA* cells. The insertion and excision of pEK26 to generate the V583 Δ*ccpA*::*ccpA* (EK26) strain was performed as previously described (64) and confirmed by colony PCR using the primers CcpAUp and CcpADown.

### Growth assessment under nutrient-limiting conditions.

Using a single colony of each strain, liquid cultures were started in THB and grown at 37°C overnight. For growth analysis, overnight cultures were diluted 1:100 in complete defined medium (CDM) (21, 22) supplemented with a range of either glucose, mannose, fructose, or *N*-acetylglucosamine concentrations (10 mM and 100 mM). Growth was monitored for 12 h in an Infinite M200 Pro plate reader (Tecan Trading AG, Switzerland) at 37°C with orbital shaking at 250 rpm. The experiment was biologically repeated three times, which included three technical replicates each time. A similar approach was used to assess the growth of *rpoN* and *ccpA* mutant strains in drip-flow biofilm growth medium (MM9YEGC or MM9YEFC) relative to that of V583.

### Microarray analysis.

Colony biofilms (65) were grown similarly to those reported previously (66). Briefly, E. faecalis strains were grown overnight in 2 ml of CDM cultures with 100 mM glucose and 20 μM hematin added, shaking at 150 rpm at 37°C. Overnight cultures were subcultured 1:1,000 under the same growth conditions and allowed to grow to an optical density (OD) of approximately 0.2, then diluted in fresh medium to an *A*_600_ of 0.1 (approximately 10^8^ cells/ml). A 10-μl aliquot of this solution was added in three discrete spots to a 25-mm, 0.2-μm-pore polycarbonate membrane affixed to the surface of CDM plus 1% agarose solid medium in a 100-mm petri dish. Plates were incubated for ∼16 h at 37°C, and polycarbonate membranes were moved to unoccupied areas of the dish and further incubated at 37°C for an additional 4 h. Membranes were then transferred to 1.5-ml microcentrifuge tubes containing 1 ml of RNAlater (Ambion) and vortexed until no visible cells remained attached to the membrane surface. Cells were pelleted by centrifugation (2 min at 10,000 × *g*), and supernatants and cell pellets were stored for RNA purification. RNA purification and Affymetrix microarray preparations were performed as described elsewhere (66, 67).

Array analyses were performed using RMA analysis through the University of Oklahoma Bioinformatics Core Facility (http://www.ou.edu/microarray/). 2σ analysis was used to determine the significant difference of fold change with a 95% confidence interval. Significant fold changes were considered for values greater than 3.4-fold for the *rpoN* mutant versus wild-type and greater than 1.8-fold for the complement versus wild-type comparisons.

### *In silico* analysis of identified proteins.

Signal peptide sequences were predicted using SignalP 5.0 and SignalP-HMM algorithms (http://www.cbs.dtu.dk/services/SignalP-5.0/) (68). Transmembrane domains were predicted using TMHMM (hidden Markov model) (http://www.cbs.dtu.dk/services/TMHMM/) (69) and TMpred (transmembrane helix propensity scale) (https://embnet.vital-it.ch/software/TMPRED_form.html) (70). LPXTG motifs were identified using PHI-BLAST and the pattern query for the sortase cell wall-sorting signal (L-P-[SKTAQEHLDN]-[TA]-[GN]-[EDASTV]) (25) with the E. faecalis collagen adhesion protein, Ace, as a template query (26). Lipoprotein predictions were conducted using Pred-Lipo software (http://www.compgen.org/tools/PRED-LIPO) (71). Default settings for Gram-positive bacteria were used in all cases.

### Quantitative real-time PCR.

Synthesis of cDNA was performed using SuperScript III reverse transcriptase (Life Technologies) from 1 μg of DNase-treated (Ambion Turbo DNase) RNA templates following the manufacturer’s instructions (Zymo Research). Random hexamer primers (Invitrogen) were used in the initial synthesis reaction. The primers used in quantitative real-time PCR (qRT-PCR) analysis are listed in Table S2. The qRT-PCR was performed with 1 μg of prepared cDNA and 300 nM each primer using PowerUp SYBR green master mix (Thermo Fisher Scientific) on a QuantStudio 3 real-time PCR system (Thermo Fisher Scientific). Following denaturation at 95°C for 3 min, the qRT-PCR was set for 50 cycles with 95°C for 10 s, 60°C for 20 s, and 72°C for 10 s. Differential gene expression was calculated using the threshold cycle (ΔΔ*C_T_*) method using the threshold cycle values for the gene of interest (*ef0019*, *ef2223*, *ef0891*, *ef1017*, *ef3210*, and *ef0255*) and the endogenous control (*ef0005* [*gyrB*]). Each qRT-PCR experiment was repeated with three biological replicates.

### Biofilm formation assessment using a drip-flow biofilm reactor.

A drip-flow biofilm reactor (DFBR) was utilized as previously described (28, 29) to assess V583, Δ*rpoN*, Δ*rpoN*::*rpoN*, Δ*ccpA*, and Δ*ccpA*::*ccpA* strains for their ability to form biofilms. Briefly, the channels of the growth chamber of the DFBR were seeded with diluted overnight cultures grown in THB (1:100) in either MM9YEGC (G = 15 mM glucose) or MM9YEFC (F = 15 mM fructose) medium and incubated at 37°C for 8 h to allow initial adherence. Subsequently, 0.1× MM9YEGC or MM9YEFC medium was fed into the 10°-tilted growth chamber of the DFBR by inlet valves and tubing at 125 μl/min for 72 h. Biofilm enumeration was conducted, aseptically, by removing the glass slides and scraping the biofilm into a 50-ml conical test tube containing 5 ml 1× phosphate-buffered saline (PBS). Homogenization of the biofilm was conducted using a Tissue-Tearor homogenizer (BioSpec Products) with a 15-s pulse, followed by serial dilution and plating on THB plates. The experiment was performed with three biological replicates.

### Animal models.

All of the procedures in the rabbit model to study experimental endocarditis and murine model for catheter-associated urinary tract infection were performed in compliance with the Animal Welfare Act and other federal statutes and regulations relating to animals and experiments involving animals. All animal protocols were approved by the Institutional Animal Care and Use Committee for Kansas State University (IACUC 3043, rabbit endocarditis) and (IACUC 3267, murine CAUTI).

### Experimental endocarditis and determination of bacterial burden.

Left-sided endocarditis was induced in New Zealand White rabbits (Charles River Laboratories International, Inc.) by introducing a polyethylene catheter with an internal diameter of 0.86 mm (Becton, Dickinson, MD), followed by injection of bacterial cultures (10^7^ CFU; E. faecalis V583 [72] and Δ*rpoN* [6] strains) via marginal ear vein after 24 h of catheterization, as previously described (30). In preparation for injections, bacterial cultures grown to the stationary phase were washed twice and diluted to achieve a concentration of 10^7^ CFU/ml in sterile saline. Groups of eight rabbits were injected with each bacterial strain (V583 and Δ*rpoN*), and two negative controls were injected with sterile saline. The rabbits were monitored for 48 h after bacterial inoculation and euthanized by intraperitoneal administration of sodium pentobarbital. Immediately after euthanasia, a cardiac stick was performed to determine bacterial CFU in blood at the time of sacrifice. Bacterial burden in the heart, liver, spleen, and kidneys was assessed by plate count, following a previously described protocol (30), and expressed as log_10_ CFU/g of tissue.

### Murine model for catheter-associated urinary tract infection.

The catheter-associated urinary tract infection (CAUTI) model used 6- to 7-week-old female wild-type C57BL/6 mice. The mice were anesthetized by isoflurane inhalation, and a 5- to 6-mm platinum-cured silicone implant tube (Renasil Sil025; Braintree Scientific, Inc.) was transurethrally placed in the urinary bladder of each mouse as previously described (31). Postimplantation, the mice were injected with 50-μl inocula of either sterile PBS or bacterial suspension (∼2 × 10^7^ CFU) by transurethral catheterization. The mice were monitored for 48 h post implantation and infection. They were euthanized by cervical dislocation after inhalation of isoflurane. To determine the degree of infection, kidneys and bladder were harvested aseptically, and their bacterial burden was determined. Also, the silicone implant tubing was retrieved from the bladder and the bacterial burden enumerated using THB medium.

### Bioinformatics and statistical analysis.

Catabolite-responsive element (*cre*) sites were identified upstream of the differentially expressed genes using the pattern analysis option in Regulatory Sequence Analysis Tools (http://rsat.ulb.ac.be/rsat/) and the *cre* consensus from Schumacher et al. (13) (WTGNNARCGNWWWCAW), as well as that from Miwa et al. (WTGWAARCGYWWWCW) (12), allowing for a 1-bp mismatch. The statistical analysis of the various growth curves performed were measured using a one-way analysis of variance (ANOVA) test. The statistical analysis of the bacterial burden determined in the various organs in the endocarditis study and CAUTI was performed using GraphPad Prism 5 software (San Diego, CA). Statistical significance was measured using a nonparametric *t* test (Mann-Whitney test).

### Data availability.

Raw array data have been deposited in the NCBI Gene Expression Omnibus (https://www.ncbi.nlm.nih.gov/geo/) under accession number GSE40237.
