# Supplementary material for: Influence of the Alternative Sigma Factor RpoN on Global Gene Expression and Carbon Catabolism in Enterococcus faecalis V583
Source: mBio. 2021 May 18;12(3):e00380-21. doi: 10.1128/mBio.00380-21 (PMC8262876; doi:10.1128/mBio.00380-21)
Supplement: FIG S1 [file mbio.00380-21-sf001.docx]

**Figure S1**

Growth of *E. faecalis* in CDM supplemented with 10mM glucose or mannose. Each graph is the average of three biological replicates, with three internal replicates each time (n=9) with standard error of the mean shown. The growth curves are represented as black (V583), blue (Δ*rpoN*), green (Δ*mptR*), red (Δ*mpoR*), yellow (Δ*mphR*), purple (Δ*lpoR*), pink (Δ*xpoABDC*), and orange (*ΔmptBACD*).
