# Supplementary material for: Influence of the Alternative Sigma Factor RpoN on Global Gene Expression and Carbon Catabolism in Enterococcus faecalis V583
Source: mBio. 2021 May 18;12(3):e00380-21. doi: 10.1128/mBio.00380-21 (PMC8262876; doi:10.1128/mBio.00380-21)
Supplement: FIG S2 [file mbio.00380-21-sf002.docx]

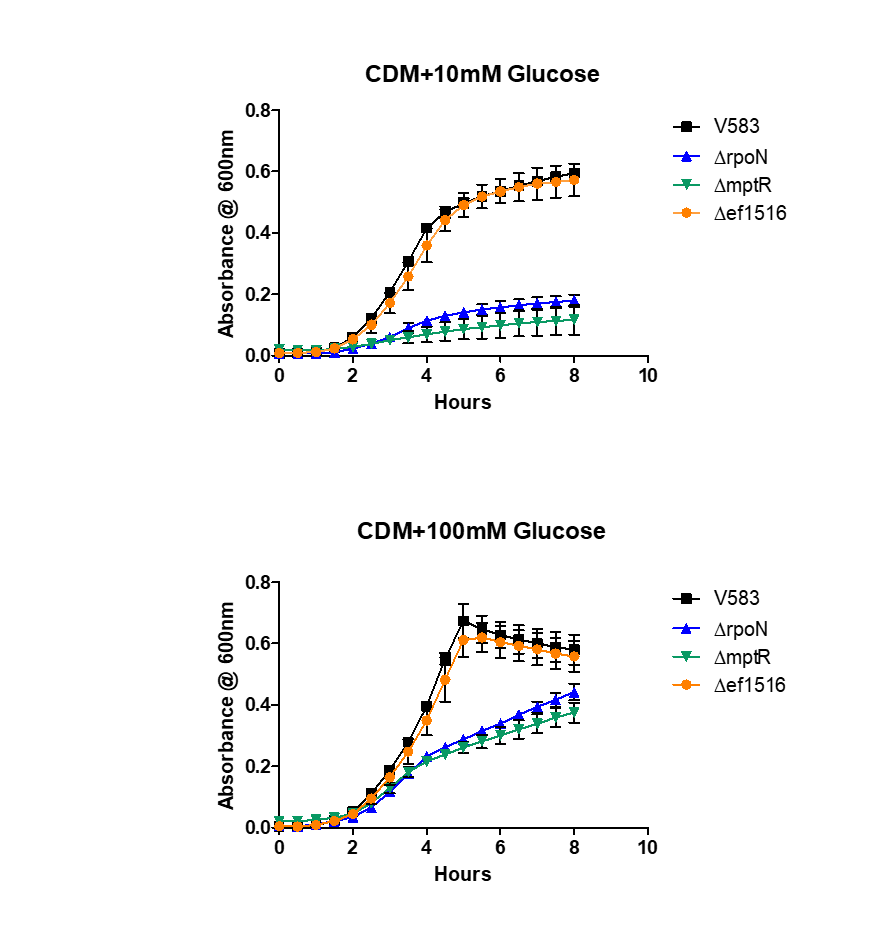


**Figure S2**

Growth of *E. faecalis* activator mutants in CDM containing 10mM or 100mM glucose. Each graph is the average of three internal replicates, repeated thrice with standard error of the mean shown. The growth curves are represented as black (V583), blue (Δ*rpoN*), green (Δ*mptR*), and orange (Δ*ef1516*).
