# Supplementary material for: Influence of the Alternative Sigma Factor RpoN on Global Gene Expression and Carbon Catabolism in Enterococcus faecalis V583
Source: mBio. 2021 May 18;12(3):e00380-21. doi: 10.1128/mBio.00380-21 (PMC8262876; doi:10.1128/mBio.00380-21)
Supplement: TABLE S3 [file mbio.00380-21-st003.docx]

Table S3. Plasmids used in this study.

| **Plasmid** | **Description** | **Reference** |
| --- | --- | --- |
| pLT06 | Markerless exchange vector; chloramphenicol resistance | ([62](#_ENREF_62)) |
| pKS200 | pLT06 + engineered *ccpA* deletion | This study |
| pMG07 | pLT06 + engineered *mptR* deletion | This study |
| pMG08 | pLT06 + engineered *lpoR* deletion | This study |
| pMG09 | pLT06 + engineered *mpoR* deletion | This study |
| pMG10 | pLT06 + engineered *mphR* deletion | This study |
| pKC02 | pLT06 + engineered *xpoABDC* deletion | This study |
| pIH03 | pLT06 + engineered *mptBACD* deletion | This study |
| pEK26 | pLT06 + engineered *ccpA* complement | This study |
| pZA13 | pLT06 + engineered *ef2223-21* deletion | This study |
| pEK04 | pLT06 + engineered *ef1516* deletion | This study |
