# Supplementary material for: Influence of the Alternative Sigma Factor RpoN on Global Gene Expression and Carbon Catabolism in Enterococcus faecalis V583
Source: mBio. 2021 May 18;12(3):e00380-21. doi: 10.1128/mBio.00380-21 (PMC8262876; doi:10.1128/mBio.00380-21)
Supplement: TABLE S4 [file mbio.00380-21-st004.docx]

| **Table S4: Differentially expressed genes in V583Δ*rpoN* compared to V583** | | |  |
| --- | --- | --- | --- |
| **Gene** | | **Function** | **Fold Change** |
| **Former Locus Tag** | **Current Locus Tag** |  |  |
| **HYPOTHETICAL PROTEINS AND PROTEINS WITH UNKNOWN FUNCTIONS** | | | |
| EF0054 | EF_RS00250 | hypothetical protein | 58.53 |
| EF0384 | EF_RS01920 | hypothetical protein | 34.97 |
| EF0711 | EF_RS03420 | conserved hypothetical protein | 32.33 |
| EF1394 | EF_RS06735 | MOSC domain-containing protein | 29.49 |
| EF0383 | EF_RS01915 | protein FdrA conserved hypothetical protein | 29.34 |
| EF0664 | EF_RS03195 | C-GCAxxG-C-C family protein | 27.97 |
| EF1227 | EF_RS05930 | NADPH-dependent oxidoreductase | 21.74 |
| EF2570 | EF_RS12210 | selenium-dependent xanthine dehydrogenase | 21.17 |
| EF1226 | EF_RS05925 | NAD(P)H-dependent oxidoreductase | 21.02 |
| EF2566 | EF_RS12190 | sulfurtransferase-like selenium metabolism protein YedF | 19.48 |
| EF2220 | EF_RS10620 | conserved hypothetical protein | 18.53 |
| EF2236 | EF_RS10700 | conserved hypothetical protein | 18.24 |
| EF0678 | EF_RS03260 | acetyltransferase, GNAT family | 16.96 |
| EF0743 | EF_RS03555 | hypothetical protein | 16.44 |
| EF0392 | EF_RS01955 | hypothetical protein | 15.39 |
| EF2564 | EF_RS12180 | putative selenium-dependent hydroxylase accessory protein YqeC | 14.57 |
| EF2565 | EF_RS12185 | conserved hypothetical protein | 12.22 |
| EF2563 | EF_RS12175 | selenium-dependent molybdenum hydroxylase system protein | 11.38 |
| EF3142 | EF_RS14885 | 6-phosphogluconate dehydrogenase family protein | 8.83 |
| EF1237 | EF_RS05975 | endonuclease/exonuclease/phosphatase family protein | 8.26 |
| EF3105 | EF_RS14705 | hypothetical protein | 7.74 |
| EF1912 | EF_RS09170 | ROK family protein | 7.19 |
| EF0405 | EF_RS02010 | Cof-type HAD-IIB family hydrolase | 7.16 |
| EF1229 | EF_RS05940 | AP2 domain-containing protein | 6.71 |
| EF1035 | EF_RS04930 | DUF4767 domain-containing protein | 6.62 |
| EF1066 | EF_RS05155 | sugar O-acetyltransferase | 6.47 |
| EF3087 | EF_RS14625 | hypothetical protein | 6.39 |
| EF1228 | EF_RS05935 | hypothetical protein | 6.29 |
| EF0389 | EF_RS01945 | membrane protein, putative | 6.19 |
| EF1919 | EF_RS09195 | acetyltransferase, GNAT family | 5.87 |
| EF0377 | EF_RS01885 | ankyrin repeat family protein | 5.56 |
| EF2441 | EF_RS11600 | conserved hypothetical protein | 5.50 |
| EF0052 | EF_RS00240 | hypothetical protein | 5.41 |
| EF3088 | EF_RS14630 | hypothetical protein | 5.34 |
| EF1075 | EF_RS05195 | acetyltransferase, GNAT family | 5.25 |
| EF1077 | EF_RS05205 | acetyltransferase, GNAT family | 5.09 |
| EF3103 | EF_RS14695 | hypothetical protein | 5.04 |
| EF3326 | EF_RS15745 | conserved hypothetical protein | 4.85 |
| EF3102 | EF_RS14690 | hypothetical protein | 4.82 |
| EF1535 | EF_RS07400 | conserved hypothetical protein | 4.69 |
| EF1061 | EF_RS05135 | amidohydrolase family protein | 4.53 |
| EF2577 | EF_RS12240 | aspartateornithine carbamoyltransferase family protein | 4.30 |
| EF3092 | EF_RS14650 | glyoxalase family protein | 4.16 |
| EF1407 | EF_RS06795 | hypothetical protein | 4.07 |
| EF1800 | EF_RS08635 | conserved hypothetical protein | 4.01 |
| EF3032 | EF_RS14375 | photosystem I biogenesis protein BtpA | 3.77 |
| EF1062 | EF_RS05140 | amidohydrolase family protein | 3.68 |
| EF0244 | EF_RS01110 | acetyltransferase, GNAT family | 3.54 |
| EF0460 | EF_RS02265 | serine hydrolase | 3.48 |
| EF1362 | EF_RS06575 | conserved domain protein | 3.41 |
| EF1933 | EF_RS09255 | hypothetical protein | 3.39 |
| EF1512 | EF_RS07310 | transglutaminase domain-containing protein | 3.37 |
| EF0113 | EF_RS00505 | ParB N-terminal domain-containing protein | 3.35 |
| EF1322 | EF_RS06370 | conserved hypothetical protein | 3.31 |
| EF3009 | EF_RS14270 | conserved hypothetical protein | 3.20 |
| EF3007 | EF_RS14260 | chloride ion channel protein | 3.15 |
| EF1239 | EF_RS05985 | conserved hypothetical protein | 3.08 |
| EF3010 | EF_RS14275 | acyl-CoA synthetase FdrA | 3.05 |
| EF1528 | EF_RS07375 | hypothetical protein | 3.01 |
| EF3245 | EF_RS15340 | phosphatase PAP2/LCP family protein | -3.10 |
| EF2944 | EF_RS13960 | hypothetical protein | -3.10 |
| EF0708 | EF_RS03405 | conserved hypothetical protein | -3.16 |
| EF0672 | EF_RS03230 | hypothetical protein | -3.31 |
| EF0341 | EF_RS01710 | hypothetical protein | -3.33 |
| EF1841 | EF_RS08835 | HD domain-containing protein | -3.79 |
| EF1199 | EF_RS05795 | conserved hypothetical protein | -4.04 |
| EF0747 | EF_RS03575 | conserved hypothetical protein | -4.26 |
| EF2899 | EF_RS13745 | NAD(P)/FAD-dependent oxidoreductase | -4.35 |
| EF0419 | EF_RS02075 | conserved hypothetical protein | -4.54 |
| EF1258 | EF_RS06065 | hypothetical protein | -5.02 |
| EF2216 | EF_RS10600 | FUSC family protein | -5.66 |
| EF2214 | EF_RS10590 | VOC family protein | -6.21 |
| EF2215 | EF_RS10595 | SRPBCC family protein | -7.27 |
| EF2896 | EF_RS13735 | hypothetical protein | -7.39 |
| EF0083 | EF_RS00370 | hypothetical protein | -7.77 |
| EF1231 | EF_RS05945 | metallophosphoesterase | -10.70 |
| EF0802 | EF_RS03825 | hypothetical protein | -29.61 |
|  |  |  |  |
| **TRANSPORT AND BINDING PROTEINS** | | | |
| EF2223 | EF_RS10635 | ABC transporter, permease protein | 257.25 |
| EF2221 | EF_RS10625 | ABC transporter, substrate-binding protein | 128.09 |
| EF2222 | EF_RS10630 | ABC transporter, permease protein | 114.80 |
| EF0892 | EF_RS04240 | amino acid ABC transporter, ATP-binding protein | 40.84 |
| EF0893 | EF_RS04245 | amino acid ABC transporter, amino acid-bindingpermease protein | 39.81 |
| EF1927 | EF_RS09230 | glycerol uptake facilitator protein | 36.40 |
| EF0385 | EF_RS01925 | major facilitator family transporter | 27.11 |
| EF1398 | EF_RS06755 | molybdenum ABC transporter, permease protein | 26.30 |
| EF1397 | EF_RS09100 | molybdenum ABC transporter, molybdenum-binding protein | 23.85 |
| EF2234 | EF_RS10690 | sugar ABC transporter, sugar-binding protein, putative | 17.24 |
| EF0387 | EF_RS01935 | sodiumdicarboxylate symporter family protein | 16.14 |
| EF1233 | EF_RS05955 | ABC transporter, permease protein | 13.83 |
| EF1920 | EF_RS09200 | C4-dicarboxylate anaerobic carrier | 13.23 |
| EF1399 | EF_RS06760 | molybdenum ABC transporter, ATP-binding protein, putative | 13.1 |
| EF0938 | EF_RS04460 | ABC transporter, ATP-bindingTOBE domain protein | 12.53 |
| EF1234 | EF_RS05960 | ABC transporter, substrate-binding protein, putative | 12.52 |
| EF1345 | EF_RS06500 | sugar ABC transporter, sugar-binding protein | 11.76 |
| EF1344 | EF_RS06495 | sugar ABC transporter, permease protein | 10.11 |
| EF1232 | EF_RS05950 | ABC transporter, permease protein | 8.63 |
| EF1207 | EF_RS05835 | citrate carrier protein, CCS family | 8.54 |
| EF2233 | EF_RS10685 | ABC transporter, permease protein | 8.46 |
| EF2992 | EF_RS14195 | major facilitator family transporter | 7.38 |
| EF3327 | EF_RS15750 | citrate transporter | 7.22 |
| EF0556 | EF_RS02720 | xylose isomerase | 7.01 |
| EF1343 | EF_RS06490 | sugar ABC transporter, permease protein | 6.65 |
| EF1400 | EF_RS06765 | cadmium-translocating P-type ATPase | 6.43 |
| EF3104 | EF_RS14700 | ABC transporter, ATP-binding protein | 5.46 |
| EF2442 | EF_RS11605 | phosphate transporter family protein | 5.45 |
| EF3000 | EF_RS14225 | cytosinepurines, uracil, thiamine, allantoin permease family protein | 4.56 |
| EF1513 | EF_RS07315 | pheromone binding protein | 4.23 |
| EF2232 | EF_RS10680 | ABC transporter, permease protein | 4.20 |
| EF3109 | EF_RS14725 | peptide ABC transporter, ATP-binding protein | 4.07 |
| EF3108 | EF_RS14720 | peptide ABC transporter, permease protein | 3.84 |
| EF3110 | EF_RS14730 | peptide ABC transporter, ATP-binding protein | 3.53 |
| EF1060 | EF_RS05130 | pheromone binding protein | 3.49 |
| EF0063 | EF_RS00285 | pheromone binding protein, putative | 3.42 |
| EF3107 | EF_RS14715 | peptide ABC transporter, permease protein | 3.40 |
| EF3106 | EF_RS14710 | peptide ABC transporter, peptide-binding protein | 3.35 |
| EF0243 | EF_RS01105 | branched-chain amino acid transport system II carrier protein | 3.22 |
| EF0429 | EF_RS02120 | TRAP dicarboxylate transporter, DctP subunit | 3.16 |
| EF0807 | EF_RS03850 | pheromone binding protein, putative | 3.15 |
| EF2593 | EF_RS03850 | ABC transporter, ATP-bindingpermease protein | -3.05 |
| EF0569 | EF_RS02755 | potassium-transporting ATPase, subunit C | -3.16 |
| EF3069 | EF_RS14545 | formatenitrite transporter family protein | -3.16 |
| EF0804 | EF_RS03835 | amino acid ABC transporter, amino acid-binding protein | -3.19 |
| EF1198 | EF_RS05790 | permease | -3.26 |
| EF0568 | EF_RS02750 | potassium-transporting ATPase, subunit B | -3.37 |
| EF0805 | EF_RS03840 | amino acid ABC transporter, ATP-binding protein | -3.56 |
| EF3004 | EF_RS14245 | sulfate transporter familySTAS domain protein | -3.57 |
| EF1968 | EF_RS09390 | ECF transporter S component | -3.66 |
| EF0806 | EF_RS03845 | amino acid ABC transporter, permease protein | -3.72 |
| EF0420 | EF_RS02080 | drug resistance transporter, EmrBQacA family protein | -3.84 |
| EF0567 | EF_RS02745 | potassium-transporting ATPase, subunit A | -3.95 |
| EF1304 | EF_RS06285 | magnesium-translocating P-type ATPase | -4.12 |
| EF0635 | EF_RS03060 | amino acid permease family protein | -5.11 |
| EF1053 | EF_RS05100 | ABC transporter, ATP-binding protein | -5.23 |
| EF0636 | EF_RS03065 | Na+H+ antiporter | -6.49 |
| EF0082 | EF_RS00365 | major facilitator family transporter | -6.82 |
| EF1814 | EF_RS08700 | drug resistance transporter, EmrBQacA family protein | -8.11 |
| EF1192 | EF_RS05760 | aquaporin Z | -11.87 |
| EF1054 | EF_RS05105 | ABC transporter, permease protein | -17.50 |
|  |  |  |  |
| **CELL ENVELOPE/SECRETED** | | | |
| EF0362 | EF_RS01815 | chitin binding protein, putative | 83.75 |
| EF0361 | EF_RS01810 | chitinase, family 2 | 69.31 |
| EF2863 | EF_RS13555 | endo-beta-N-acetylglucosaminidase | 62.34 |
| EF0713 | EF_RS03425 | WxL domain-containing protein | 58.00 |
| EF0114 | EF_RS00510 | glycosyl hydrolase, family 20 | 48.20 |
| EF0714 | EF_RS03430 | WxL domain-containing protein | 29.26 |
| EF0108 | EF_RS00480 | YfcC family protein | 13.04 |
| EF2237 | EF_RS10705 | lipoprotein, putative | 7.17 |
| EF1657 | EF_RS07965 | membrane protein, putative | 5.80 |
| EF0062 | EF_RS00280 | LPXTG cell wall anchor domain-containing protein | 4.40 |
| EF0673 | EF_RS03235 | membrane protein, putative | 4.05 |
| EF2662 | EF_RS12620 | choline binding protein | 3.50 |
| EF2627 | EF_RS12460 | teichoic acid glycosylation protein, putative | -3.11 |
| EF1340 | EF_RS06475 | pheromone cAM373 precursor lipoprotein | -3.16 |
| EF2746 | EF_RS13000 | dltD protein | -3.45 |
| EF0468 | EF_RS02305 | LemA family protein | -3.71 |
| EF2749 | EF_RS13015 | D-alanine-activating enzyme, putative | -3.83 |
| EF2748 | EF_RS13010 | basic membrane protein DtlB | -4.01 |
| EF2747 | EF_RS13005 | D-alanyl carrier protein | -4.07 |
| EF0443 | EF_RS02180 | LysM peptidoglycan-binding domain-containing protein | -4.25 |
| EF2750 | EF_RS13020 | teichoic acid D-Ala incorporation-associated protein DltX | -4.95 |
| EF0746 | EF_RS03570 | penicillin-binding protein, putative | -11.48 |
|  |  |  |  |
| **ENERGY METABOLISM** | | | |
| EF1929 | EF_RS09240 | glycerol kinase | 53.22 |
| EF1928 | EF_RS09235 | alpha-glycerophosphate oxidase | 43.44 |
| EF0253 | EF_RS01155 | aldehyde dehydrogenase | 35.88 |
| EF2562 | EF_RS12170 | flavodoxin | 19.95 |
| EF0106 | EF_RS00470 | carbamate kinase | 19.07 |
| EF1068 | EF_RS05160 | aldose 1-epimerase | 18.97 |
| EF0677 | EF_RS03255 | phosphoglucomutasephosphomannomutase family protein | 18.13 |
| EF0386 | EF_RS01930 | carbamate kinase | 16.9 |
| EF0104 | EF_RS00460 | arginine deiminase | 15.83 |
| EF1661 | EF_RS07985 | branched-chain alpha-keto acid dehydrogenase, E3 component, dihydrolipoamide dehydrogenase | 14.95 |
| EF1658 | EF_RS07970 | branched-chain alpha-keto acid, E2 component, dihydrolipoamide acetyltransferase | 14.28 |
| EF3135 | EF_RS14850 | mannonate dehydratase, putative | 13.37 |
| EF3134 | EF_RS14845 | 2-dehydro-3-deoxyphosphogluconate aldolase4-hydroxy-2-oxoglutarate aldolase | 10.43 |
| EF2559 | EF_RS12155 | pyruvate flavodoxinferredoxin oxidoreductase family protein | 9.99 |
| EF0388 | EF_RS01940 | ureidoglycolate dehydrogenase | 9.27 |
| EF1659 | EF_RS07975 | branched-chain alpha-keto acid dehydrogenase, E1 component, beta subunit | 8.46 |
| EF1349 | EF_RS06515 | glycosyl hydrolase, family 13 | 7.96 |
| EF2235 | EF_RS10695 | glucuronyl hydrolase, putative | 7.81 |
| EF0390 | EF_RS01950 | amidohydrolase family protein | 7.61 |
| EF1824 | EF_RS08745 | glycosyl hydrolase, family 31fibronectin type III domain protein | 6.82 |
| EF0900 | EF_RS04270 | aldehyde-alcohol dehydrogenase | 6.69 |
| EF1206 | EF_RS05830 | malate dehydrogenase, decarboxylating | 6.26 |
| EF2579 | EF_RS12250 | diaminopropionate ammonia-lyase, putative | 6.21 |
| EF1236 | EF_RS05970 | acetyl xylan esterase, putative | 5.82 |
| EF3325 | EF_RS15740 | sodium ion-translocating decarboxylase, biotin carboxyl carrier protein | 5.29 |
| EF1347 | EF_RS06505 | glycosyl hydrolase, family 13 | 5.18 |
| EF1660 | EF_RS07980 | branched-chain alpha-keto acid dehydrogenase, E1 component, alpha subunit | 5.14 |
| EF1662 | EF_RS07990 | butyrate kinase | 5.13 |
| EF1503 | EF_RS07270 | fructose-1,6-bisphosphatase | 4.95 |
| EF1805 | EF_RS08660 | glycosyl hydrolase, family 35 | 4.81 |
| EF2268 | EF_RS10830 | alginate lyase family protein | 4.73 |
| EF2440 | EF_RS11595 | ChbG/HpnK family deacetylase | 4.71 |
| EF1360 | EF_RS06565 | dihydroxyacetone kinase subunit DhaK | 4.70 |
| EF2575 | EF_RS12235 | carbamate kinase | 4.52 |
| EF2581 | EF_RS12260 | putative selenate reductase subunit YgfK | 4.41 |
| EF0271 | EF_RS01375 | glycosyl hydrolase, family 1 | 4.33 |
| EF2996 | EF_RS14210 | (S)-ureidoglycine aminohydrolase | 4.13 |
| EF0291 | EF_RS01465 | glycosyl hydrolase, family 1 | 4.06 |
| EF0551 | EF_RS02695 | glycosyl hydrolase, family 31 | 3.89 |
| EF2265 | EF_RS10815 | carbohydrate kinase, pfkB family | 3.88 |
| EF2264 | EF_RS10810 | 4-deoxy-l-threo-5-hexosulose-uronate ketol-isomerase | 3.87 |
| EF1361 | EF_RS06570 | dihydroxyacetone kinase subunit L | 3.84 |
| EF1238 | EF_RS05980 | glycosyl hydrolase, family 3 | 3.79 |
| EF3157 | EF_RS14945 | glycosyl hydrolase, family 65 | 3.78 |
| EF0459 | EF_RS02260 | N-acetylmuramic acid 6-phosphate etherase | 3.77 |
| EF2272 | EF_RS10850 | glucuronyl hydrolase, putative | 3.74 |
| EF3158 | EF_RS14950 | beta-phosphoglucomutase | 3.69 |
| EF1348 | EF_RS06510 | glucan 1,6-alpha-glucosidase, putative | 3.61 |
| EF2709 | EF_RS12840 | glycosyl hydrolase, family 2 | 3.56 |
| EF1158 | EF_RS05600 | N4-(beta-N-acetylglucosaminyl)-L-asparaginase, putative | 3.52 |
| EF1071 | EF_RS05175 | galactose-1-phosphate uridylyltransferase | 3.33 |
| EF2646 | EF_RS12545 | glycerate kinase, putative | 3.32 |
| EF1069 | EF_RS05165 | galactokinase | 3.17 |
| EF1707 | EF_RS08205 | alpha-mannosidase | 3.07 |
| EF2722 | EF_RS12890 | L-serine dehydratase, iron-sulfur-dependent, alpha subunit | -3.19 |
| EF2500 | EF_RS11875 | glycine cleavage system protein H | -3.69 |
| EF2721 | EF_RS12885 | L-serine dehydratase, iron-sulfur-dependent, beta subunit | -3.77 |
|  |  |  |  |
| **PTS SYSTEMS** | | | |
| EF2213 | EF_RS10585 | PTS system, IIBC components | 23.3 |
| EF3138 | EF_RS14865 | PTS system, IID component | 17.41 |
| EF3137 | EF_RS14860 | PTS system, IIB component | 16.00 |
| EF1516 | EF_RS07325 | PTS system, IIABC components | 14.22 |
| EF3136 | EF_RS14855 | PTS system, IIA component | 12.08 |
| EF0553 | EF_RS02705 | PTS system, IID component | 11.35 |
| EF0554 | EF_RS02710 | PTS system, IIB component | 9.6 |
| EF1529 | EF_RS07380 | PTS system, IIC component, putative | 8.56 |
| EF1803 | EF_RS08650 | PTS system, IIC component | 8.28 |
| EF3139 | EF_RS14870 | PTS system, IIC component | 7.55 |
| EF1802 | EF_RS08645 | PTS system, IID component | 7.35 |
| EF3031 | EF_RS14370 | PTS system, IIB component | 7.03 |
| EF0270 | EF_RS01370 | PTS system, beta-glucoside-specific IIABC component | 6.91 |
| EF0292 | EF_RS01470 | PTS system, IIC component | 6.62 |
| EF1804 | EF_RS08655 | PTS system, IIB component | 6.36 |
| EF0552 | EF_RS02700 | PTS system, IIC component | 6.21 |
| EF1836 | EF_RS08810 | PTS system, IIA component, putative | 5.84 |
| EF1837 | EF_RS08815 | PTS system, IIB component, putative | 5.74 |
| EF0816 | EF_RS03890 | PTS system, IIC component | 5.32 |
| EF1359 | EF_RS06560 | PTS-dependent dihydroxyacetone kinase phosphotransferase subunit DhaM | 4.70 |
| EF3030 | EF_RS14365 | PTS system, IIC component | 4.58 |
| EF1801 | EF_RS08640 | PTS system, IIA component | 4.42 |
| EF3029 | EF_RS14360 | PTS system, IID component | 4.32 |
| EF2269 | EF_RS10835 | PTS system, IID component | 4.14 |
| EF0958 | EF_RS04555 | PTS system, IIABC components | 3.91 |
| EF0815 | EF_RS03885 | PTS system, IIAB components | 3.81 |
| EF0817 | EF_RS03895 | PTS system, IID component | 3.77 |
| EF0555 | EF_RS02715 | PTS system, IIA component | 3.75 |
| EF0456 | EF_RS02245 | PTS system, IID component | 3.75 |
| EF2270 | EF_RS10840 | PTS system, IIC component | 3.59 |
| EF2267 | EF_RS10825 | PTS system, IIA component | 3.42 |
| EF1018 | EF_RS04845 | PTS system, IIA component | -3.05 |
| EF1019 | EF_RS04850 | PTS system, IIC component | -4.12 |
| EF3213 | EF_RS15200 | PTS system, IID component | -4.3 |
| EF1017 | EF_RS04840 | PTS system, IIB component | -4.49 |
| EF3212 | EF_RS15195 | PTS system, IIC component | -5.34 |
| EF3210 | EF_RS15185 | PTS system, IIA component, putative | -6.27 |
| EF3211 | EF_RS15190 | PTS system, IIB component | -6.57 |
| EF0020 | EF_RS00090 | PTS system, mannose-specific IIAB components | -90.38 |
| EF0021 | EF_RS00095 | PTS system, mannose-specific IIC component | -100.36 |
| EF0022 | EF_RS00100 | PTS system, mannose-specific IID component | -107.49 |
| EF0019 | EF_RS00085 | PTS system, IIB component | -152.11 |
|  |  |  |  |
| **REGULATORY FUNCTIONS** | | | |
| EF0107 | EF_RS00475 | transcriptional regulator, CrpFnr family | 18.06 |
| EF1515 | EF_RS07320 | transcription antiterminator, bglG family | 14.09 |
| EF0432 | EF_RS02135 | transcriptional regulator, AraC family | 12.37 |
| EF1656 | EF_RS07960 | transcriptional regulator, LysR family | 8.75 |
| EF1591 | EF_RS07670 | transcriptional regulator, AraC family | 8.39 |
| EF3144 | EF_RS14890 | phosphosugar-binding transcriptional regulator, RpiR family | 8.25 |
| EF2711 | EF_RS12850 | transcriptional regulator, AraC family | 7.88 |
| EF0103 | EF_RS00455 | transcriptional regulator, ArgR family | 5.68 |
| EF3008 | EF_RS14265 | PucR family transcriptional regulator | 3.56 |
| EF0102 | EF_RS00450 | transcriptional regulator, ArgR family | 3.33 |
| EF0382 | EF_RS01910 | PucR family transcriptional regulator | 3.32 |
| EF3328 | EF_RS15755 | transcriptional regulator, GntR family | 3.07 |
| EF0676 | EF_RS03250 | arginine repressor | 3.05 |
| EF2933 | EF_RS13905 | redox-sensing transcriptional repressor Rex | 3.03 |
| EF2594 | EF_RS12320 | transcriptional regulator, TetR family | -3.45 |
| EF2703 | EF_RS12815 | transcriptional regulator | -4.07 |
| EF1302 | EF_RS06275 | transcriptional regulator, putative | -4.41 |
| EF1224 | EF_RS05915 | transcriptional regulator, CroCI family | -4.56 |
| EF1303 | EF_RS06280 | transcriptional regulator, LysR family | -4.92 |
| EF0782 | EF_RS03735 | RNA polymerase sigma-54 factor | -132.88 |
|  |  |  |  |
| **CELLULAR PROCESSES** | | | |
| EF2569 | EF_RS12205 | molybdenum cofactor cytidylyltransferase | 21.29 |
| EF0439 | EF_RS02170 | immunity protein PlnM, putative | 11.35 |
| EF3023 | EF_RS14340 | polysaccharide lyase, family 8 | 8.85 |
| EF1076 | EF_RS05200 | streptomycin 3-adenylyltransferase, putative | 5.84 |
| EF0818 | EF_RS03900 | polysaccharide lyase, family 8 | 3.61 |
| EF1502 | EF_RS07265 | beta-lactamase, putative | 3.25 |
| EF1959 | EF_RS09350 | toll/interleukin-1 receptor domain-containing protein | 3.23 |
| EF2739 | EF_RS12970 | alkyl hydroperoxide reductase, C subunit | -3.04 |
| EF1300 | EF_RS06265 | cell division protein, FtsWRodASpovE family | -3.69 |
| EF1301 | EF_RS06270 | cell division protein, FtsWRodASpovE family | -3.89 |
|  |  |  |  |
| **AMINO ACID BIOSYNTHESIS** | | | |
| EF0891 | EF_RS04235 | aspartate aminotransferase, putative | 47.93 |
| EF0105 | EF_RS00465 | ornithine carbamoyltransferase | 19.35 |
| EF2568 | EF_RS12200 | aminotransferase, class V | 18.70 |
| EF2560 | EF_RS12160 | glutamate synthase (NADPH), homotetrameric | 10.5 |
| EF2994 | EF_RS14200 | alanine--glyoxylate aminotransferase family protein | 3.41 |
|  |  |  |  |
| **TWO COMPONENT SYSTEMS** | | | |
| EF2218 | EF_RS10610 | DNA-binding response regulator, AraC family | 12.18 |
| EF2219 | EF_RS10615 | sensor histidine kinase | 11.03 |
|  |  |  |  |
| **BIOSYNTHESIS OF COFACTORS,PROSTHETIC GROUPS AND CARRIERS** | | | |
| EF1395 | EF_RS06740 | molybdenum cofactor biosynthesis family protein | 38.35 |
| EF1396 | EF_RS06745 | molybdenum cofactor biosynthesis family protein, putative | 35.07 |
| EF1393 | EF_RS06730 | molybdopterin cofactor biosynthesis protein A, putative | 30.09 |
| EF1225 | EF_RS05920 | thiamin biosynthesis ApbE, putative | 23.88 |
| EF1392 | EF_RS06725 | molybdenum cofactor biosynthesis protein MoaC | 4.44 |
| EF1655 | EF_RS07955 | 2-dehydropantoate 2-reductase, putative | 4.3 |
| EF1391 | EF_RS06720 | molybdenum cofactor biosynthesis family protein | 3.06 |
| EF1969 | EF_RS09395 | phosphomethylpyrimidine kinase, putative | -3.38 |
| EF2445 | EF_RS11620 | 2-dehydropantoate 2-reductase, putative | -4.14 |
|  |  |  |  |
| **DNA METABOLISM** | | | |
| EF0053 | EF_RS00245 | DNA polymerase III, epsilon subunit | 7.74 |
|  |  |  |  |
| **TRANSCRIPTION** | | | |
| EF0115 | EF_RS00515 | endoribonuclease L-PSP, putative | 30.17 |
| EF3214 | EF_RS15205 | ATP-dependent helicase, DEAH-box family, putative | -3.63 |
|  |  |  |  |
| **PRYRIMIDINE RIBONUCLEOTIDE BIOSYNTHESIS** | | | |
| EF2561 | EF_RS12165 | dihydroorotate dehydrogenase electron transfer subunit, putative | 13.65 |
| EF2999 | EF_RS14220 | allantoinase, putative | 5.82 |
|  |  |  |  |
| **NUCLEOTIDE RELATED** | | | |
| EF1921 | EF_RS09205 | inosine-uridine preferring nucleoside hydrolase | 15.75 |
| EF1036 | EF_RS04935 | nucleoside diphosphate kinase | 11.54 |
| EF2580 | EF_RS12255 | D-hydantoinase | 6.39 |
| EF1958 | EF_RS09345 | deoxyguanosinetriphosphate triphosphohydrolase, putative | 3.61 |
|  |  |  |  |
| **CENTRAL INTERMEDIARY METABOLISM** | | | |
| EF3141 | EF_RS14880 | D-isomer specific 2-hydroxyacid dehydrogenase family protein | 16.2 |
| EF0895 | EF_RS04250 | glycerol dehydrogenase, putative | 15.54 |
| EF3140 | EF_RS14875 | alcohol dehydrogenase, iron-containing | 9.99 |
| EF1358 | EF_RS06555 | glycerol dehydrogenase, putative | 4.88 |
| EF1364 | EF_RS06585 | acetyl-CoA acetyltransferasehydroxymethylglutaryl-CoA reductase, degradative | -3.41 |
| EF1813 | EF_RS08695 | sulfatase domain protein | -11.88 |
|  |  |  |  |
| **PHAGE PROTEINS** | | | |
| EF0354 | EF_RS01775 | holin, putative | -3.02 |
| EF0339 | EF_RS01705 | major capsid protein, putative | -3.15 |
|  |  |  |  |
| **FATTY ACID AND PHOSPHOLIPID METABOLISM** | | | |
| EF1663 | EF_RS07995 | branched-chain phosphotransacylase | 10.28 |
|  |  |  |  |
| **PROTEIN SYNTHESIS AND FATE** | | | |
| EF2567 | EF_RS12195 | selenide, water dikinase | 16.82 |
| EF2471 | EF_RS11740 | arginyl-tRNA synthetase | 7.8 |
| EF2997 | EF_RS14215 | peptidase, M20M25M40 family | 3.64 |
| EF2578 | EF_RS12245 | peptidase, M20M25M40 family | 3.02 |
| EF2858 | EF_RS13530 | threonyl-tRNA synthetase | -3.14 |
| EF3207 | EF_RS15170 | tRNA-dihydrouridine synthase | -3.64 |
| EF0633 | EF_RS03050 | tyrosyl-tRNA synthetase | -4.39 |
| EF0634 | EF_RS03055 | tyrosine decarboxylase | -4.90 |
