# Supplementary material for: Influence of the Alternative Sigma Factor RpoN on Global Gene Expression and Carbon Catabolism in Enterococcus faecalis V583
Source: mBio. 2021 May 18;12(3):e00380-21. doi: 10.1128/mBio.00380-21 (PMC8262876; doi:10.1128/mBio.00380-21)
Supplement: TABLE S5 [file mbio.00380-21-st005.docx]

| **Table S5: Putative *cre* sites in differentially expressed genes in V583Δ*rpoN* compared to V583** | | | | | | | |
| --- | --- | --- | --- | --- | --- | --- | --- |
| **Gene** | | **Function** | **Fold change** | **Start** | **End** | ***cre* Sequence (WTGNNARCGNWWWCAW)** |  |
| **Former Locus Tag** | **Current Locus Tag** |  | | | | |  |
| **Upregulated** | |  |  |  |  |  |  |
| EF0052 | EF_RS00240 | hypothetical protein | 5.41 |  |  |  |  |
| EF0053 | EF_RS00245 | DNA polymerase III, epsilon subunit | 7.74 |  |  |  |  |
| EF0054 | EF_RS00250 | hypothetical protein | 58.53 | -92 | -78 | TCGAAAGCGCTTTCT |  |
| EF0104 | EF_RS00460 | arginine deiminase | 15.83 | -146 | -132 | ATGAAAGCGCATTCT |  |
| EF0105 | EF_RS00465 | ornithine carbamoyltransferase | 19.35 |  |  |  |  |
| EF0106 | EF_RS00470 | carbamate kinase | 19.07 |  |  |  |  |
| EF0107 | EF_RS00475 | transcriptional regulator, CrpFnr family | 18.06 |  |  |  |  |
| EF0108 | EF_RS00480 | C4-dicarboxylate transporter, putative | 13.04 |  |  |  |  |
| EF0114 | EF_RS00510 | glycosyl hydrolase, family 20 | 48.20 | -35 | -21 | GTGTATGCGCTTTCT |  |
| EF0115 | EF_RS00515 | endoribonuclease L-PSP, putative | 30.17 | -49 | -35 | ATGTAAGCGGAATCA |  |
| EF0253 | EF_RS01155 | aldehyde dehydrogenase | 35.88 | -38 | -24 | TTGTAAGCGGATACA |  |
| EF0291 | EF_RS01465 | glycosyl hydrolase, family 1 | 4.06 |  |  |  |  |
| EF0292 | EF_RS01470 | PTS system, IIC component | 6.62 | -51 | -37 | ATGTAAACGGATACA |  |
| EF0361 | EF_RS01810 | chitinase, family 2 | 69.31 |  |  |  |  |
| EF0362 | EF_RS01815 | chitin binding protein, putative | 83.75 | -41 | -27 | CTGTAAGCGCATACA |  |
| EF0382 | EF_RS01910 | PucR family transcriptional regulator | 3.32 | -76 | -62 | ATGAAAACACTTTCT |  |
| EF0383 | EF_RS01915 | protein FdrA conserved hypothetical protein | 29.34 | -61 | -47 | ATGAAAACACTTTCT |  |
| EF0405 | EF_RS02010 | hydrolase, haloacid dehalogenase-like family | 7.16 | -71 | -57 | ATGTAAACGGATTCT |  |
| EF0439 | EF_RS02170 | immunity protein PlnM, putative | 13.54 | -49 | -35 | ATGAAAACGTTATCA |  |
| EF0551 | EF_RS02695 | glycosyl hydrolase, family 31 | 3.89 | -85 | -70 | ATACAAACGCTTTCAT |  |
| EF0552 | EF_RS02700 | PTS system, IIC component | 6.21 |  |  |  |  |
| EF0553 | EF_RS02705 | PTS system, IID component | 11.35 |  |  |  |  |
| EF0554 | EF_RS02710 | PTS system, IIB component | 9.6 |  |  |  |  |
| EF0555 | EF_RS02715 | PTS system, IIA component | 3.75 |  |  |  |  |
| EF0664 | EF_RS03195 | hypothetical protein | 27.97 | -17 | -3 | ATGAAAGCGGATACA |  |
| EF0938 | EF_RS04460 | ABC transporter, ATP-bindingTOBE domain protein | 12.53 | -60 | -46 | ATGAAAACGCTATCT |  |
| EF1036 | EF_RS04935 | nucleoside diphosphate kinase | 11.54 | -75 | -61 | ATGAAAGCGGATACT |  |
| EF1066 | EF_RS05155 | hexapeptide-repeat containing-acetyltransferase | 6.47 |  |  |  |  |
| EF1068 | EF_RS05160 | aldose 1-epimerase | 18.97 | -126 | -112 | TTGAAAACGTGTACA |  |
| EF1069 | EF_RS05165 | galactokinase | 3.17 | -109 | -95 | TTGTACACGTTTTCA |  |
| EF1158 | EF_RS05600 | N4-(beta-N-acetylglucosaminyl)-L-asparaginase, putative | 3.52 | -61 | -46 | TCGTTAACGCTTACAT |  |
| EF1206 | EF_RS05830 | malate dehydrogenase, decarboxylating | 6.26 |  |  |  |  |
| EF1207 | EF_RS05835 | citrate carrier protein, CCS family | 8.54 | -53 | -39 | ATGTAAACGTTTTCT |  |
| EF1232 | EF_RS05950 | ABC transporter, permease protein | 8.63 | -81 | -67 | ATGTAAGGGTTTACA |  |
| EF1233 | EF_RS05955 | ABC transporter, permease protein | 13.83 |  |  |  |  |
| EF1234 | EF_RS05960 | ABC transporter, substrate-binding protein, putative | 12.52 |  |  |  |  |
| EF1358 | EF_RS06555 | glycerol dehydrogenase, putative | 4.88 | -51 | -37 | ATGAAAGCGTTTTAT |  |
| EF1359 | EF_RS06560 | PTS-dependent dihydroxyacetone kinase phosphotransferase subunit DhaM | 4.70 |  |  |  |  |
| EF1360 | EF_RS06565 | dihydroxyacetone kinase subunit DhaK | 4.70 |  |  |  |  |
| EF1361 | EF_RS06570 | dihydroxyacetone kinase subunit L | 3.84 |  |  |  |  |
| EF1392 | EF_RS06725 | molybdenum cofactor biosynthesis protein MoaC | 4.44 | -38 | -24 | GTGTAAACGTTAACA |  |
| EF1393 | EF_RS06730 | molybdopterin cofactor biosynthesis protein A, putative | 30.09 |  |  |  |  |
| EF1394 | EF_RS06735 | conserved hypothetical protein | 29.49 |  |  |  |  |
| EF1395 | EF_RS06740 | molybdenum cofactor biosynthesis family protein | 38.35 |  |  |  |  |
| EF1396 | EF_RS06745 | molybdenum cofactor biosynthesis family protein, putative | 35.07 |  |  |  |  |
| EF1397 | EF_RS09100 | molybdenum ABC transporter, molybdenum-binding protein | 23.85 |  |  |  |  |
| EF1398 | EF_RS06755 | molybdenum ABC transporter, permease protein | 26.3 |  |  |  |  |
| EF1399 | EF_RS06760 | molybdenum ABC transporter, ATP-binding protein, putative | 13.1 |  |  |  |  |
| EF1400 | EF_RS06765 | cadmium-translocating P-type ATPase | 6.43 |  |  |  |  |
| EF1407 | EF_RS06795 | hypothetical protein | 4.07 | -59 | -44 | ATGATAACGATTTCTT |  |
| EF1591 | EF_RS07670 | transcriptional regulator, AraC family | 8.39 | -32 | -17 | TAGAAAGCGGATACAA |  |
| EF1656 | EF_RS07960 | transcriptional regulator, LysR family | 8.75 | -33 | -19 | TTAAAAGCGCTTACA |  |
| EF1657 | EF_RS07965 | membrane protein, putative | 5.8 |  |  |  |  |
| EF1658 | EF_RS07970 | branched-chain alpha-keto acid, E2 component, dihydrolipoamide acetyltransferase | 14.28 |  |  |  |  |
| EF1659 | EF_RS07975 | branched-chain alpha-keto acid dehydrogenase, E1 component, beta subunit | 8.46 |  |  |  |  |
| EF1660 | EF_RS07980 | branched-chain alpha-keto acid dehydrogenase, E1 component, alpha subunit | 5.14 |  |  |  |  |
| EF1661 | EF_RS07985 | branched-chain alpha-keto acid dehydrogenase, E3 component, dihydrolipoamide dehydrogenase | 14.95 |  |  |  |  |
| EF1662 | EF_RS07990 | butyrate kinase | 5.13 |  |  |  |  |
| EF1663 | EF_RS07995 | branched-chain phosphotransacylase | 10.28 | -61 | -47 | ATGTAAACGCATACA |  |
| EF1800 | EF_RS08635 | conserved hypothetical protein | 4.01 | -41 | -27 | ATGAAAGCGTGTTCA |  |
| EF1801 | EF_RS08640 | PTS system, IIA component | 4.42 |  |  |  |  |
| EF1802 | EF_RS08645 | PTS system, IID component | 7.35 |  |  |  |  |
| EF1803 | EF_RS08650 | PTS system, IIC component | 8.28 |  |  |  |  |
| EF1804 | EF_RS08655 | PTS system, IIB component | 6.36 |  |  |  |  |
| EF1805 | EF_RS08660 | glycosyl hydrolase, family 35 | 4.81 | -30 | -16 | TTGAAAGCGTTTACT |  |
| EF1824 | EF_RS08745 | glycosyl hydrolase, family 31fibronectin type III domain protein | 6.82 | -255 | -241 | ATGAAAACGCATTCA |  |
| EF1836 | EF_RS08810 | PTS system, IIA component, putative | 5.84 | -63 | -49 | TTGAAAGCGTTTTAT |  |
| EF1837 | EF_RS08815 | PTS system, IIB component, putative | 5.74 |  |  |  |  |
| EF1919 | EF_RS09195 | acetyltransferase, GNAT family | 5.87 | -76 | -61 | TTAACAGCGCTTTCAT |  |
| EF1920 | EF_RS09200 | C4-dicarboxylate anaerobic carrier | 13.23 |  |  |  |  |
| EF1921 | EF_RS09205 | inosine-uridine preferring nucleoside hydrolase | 15.75 |  |  |  |  |
| EF1927 | EF_RS09230 | glycerol uptake facilitator protein | 36.4 |  |  |  |  |
| EF1928 | EF_RS09235 | alpha-glycerophosphate oxidase | 43.44 |  |  |  |  |
| EF1929 | EF_RS09240 | glycerol kinase | 53.22 | -146  -36 | -132 | TTGAAAGCGTTGTCT |  |
|  |  |  |  |  | -22 | TTGAAATCGTTTTCT |  |
| EF2221 | EF_RS10625 | ABC transporter, substrate-binding protein | 128.09 |  |  |  |  |
| EF2222 | EF_RS10630 | ABC transporter, permease protein | 114.8 |  |  |  |  |
| EF2223 | EF_RS10635 | ABC transporter, permease protein | 257.25 | -38 | -24 | ATGAAAACGCTATTA |  |
| EF2232 | EF_RS10680 | ABC transporter, permease protein | 4.2 |  |  |  |  |
| EF2233 | EF_RS10685 | ABC transporter, permease protein | 8.46 |  |  |  |  |
| EF2234 | EF_RS10690 | sugar ABC transporter, sugar-binding protein, putative | 17.24 |  |  |  |  |
| EF2235 | EF_RS10695 | glucuronyl hydrolase, putative | 7.81 |  |  |  |  |
| EF2236 | EF_RS10700 | conserved hypothetical protein | 18.24 |  |  |  |  |
| EF2237 | EF_RS10705 | lipoprotein, putative | 7.17 | -35 | -21 | ATTAAAGCGCTTTCT |  |
| EF2559 | EF_RS12155 | pyruvate flavodoxinferredoxin oxidoreductase family protein | 9.99 | -68 | -53 | ATGTAAGGGGTTACAA |  |
| EF2560 | EF_RS12160 | glutamate synthase (NADPH), homotetrameric | 10.5 |  |  |  |  |
| EF2561 | EF_RS12165 | dihydroorotate dehydrogenase electron transfer subunit, putative | 13.65 |  |  |  |  |
| EF2562 | EF_RS12170 | flavodoxin | 19.95 |  |  |  |  |
| EF2709 | EF_RS12840 | glycosyl hydrolase, family 2 | 3.56 | -56 | -41 | TTGGAAACGATATGAA |  |
| EF2711 | EF_RS12850 | transcriptional regulator, AraC family | 7.88 | -37 | -23 | ATGTAAAGGCTTTCT |  |
| EF2863 | EF_RS13555 | endo-beta-N-acetylglucosaminidase | 62.34 | -45 | -31 | TTGTAAGCGCTAACA |  |
| EF2996 | EF_RS14210 | conserved hypothetical protein | 4.13 |  |  |  |  |
| EF2997 | EF_RS14215 | peptidase, M20M25M40 family | 3.64 |  |  |  |  |
| EF2999 | EF_RS14220 | allantoinase, putative | 5.82 |  |  |  |  |
| EF3000 | EF_RS14225 | cytosinepurines, uracil, thiamine, allantoin permease family protein | 4.56 | -118 | -104 | TTGTAAGCGCTTTTT |  |
| EF3023 | EF_RS14340 | polysaccharide lyase, family 8 | 8.85 | -209 | -195 | GTGAAAGCGTAAACA |  |
| EF3088 | EF_RS14630 | hypothetical protein | 5.34 | -58 | -44 | ATAAAAACGTTTTCT |  |
| EF3134 | EF_RS14845 | 2-dehydro-3-deoxyphosphogluconate aldolase4-hydroxy-2-oxoglutarate aldolase | 10.43 |  |  |  |  |
| EF3135 | EF_RS14850 | mannonate dehydratase, putative | 13.37 |  |  |  |  |
| EF3136 | EF_RS14855 | PTS system, IIA component | 12.08 |  |  |  |  |
| EF3137 | EF_RS14860 | PTS system, IIB component | 16 |  |  |  |  |
| EF3138 | EF_RS14865 | PTS system, IID component | 17.41 |  |  |  |  |
| EF3139 | EF_RS14870 | PTS system, IIC component | 7.55 |  |  |  |  |
| EF3140 | EF_RS14875 | alcohol dehydrogenase, iron-containing | 9.99 |  |  |  |  |
| EF3141 | EF_RS14880 | D-isomer specific 2-hydroxyacid dehydrogenase family protein | 16.2 |  |  |  |  |
| EF3142 | EF_RS14885 | 6-phosphogluconate dehydrogenase family protein | 8.83 | -68 | -34 | ATGTAAACGATTACA |  |
| EF3144 | EF_RS14890 | phosphosugar-binding transcriptional regulator, RpiR family | 8.25 | -53 | -39 | ATGAAAAGGCATTCA |  |
| EF3325 | EF_RS15740 | sodium ion-translocating decarboxylase, biotin carboxyl carrier protein | 5.29 |  |  |  |  |
| EF3326 | EF_RS15745 | conserved hypothetical protein | 4.85 | -373 | -359 | TTGTAAGCGTTAACA |  |
| EF3327 | EF_RS15750 | citrate transporter | 7.22 | -45 | -31 | TTGTAAGCGTTAACA |  |
|  |  |  |  |  |  |  |  |
| **Downregulated** | |  | | | | |  |
| EF1017 | EF_RS04840 | PTS system, IIB component | -4.49 | -173 | -158 | TTGGAAACGCACACAA |  |
| EF1018 | EF_RS04845 | PTS system, IIA component | -3.05 |  |  |  |  |
| EF1019 | EF_RS04850 | PTS system, IIC component | -4.12 |  |  |  |  |

Strongly conserved residues of the *cre* consensus sequence are underlined.
