# Supplementary material for: Influence of the Alternative Sigma Factor RpoN on Global Gene Expression and Carbon Catabolism in Enterococcus faecalis V583
Source: mBio. 2021 May 18;12(3):e00380-21. doi: 10.1128/mBio.00380-21 (PMC8262876; doi:10.1128/mBio.00380-21)
Supplement: FIG S4 [file mbio.00380-21-sf004.docx]

**
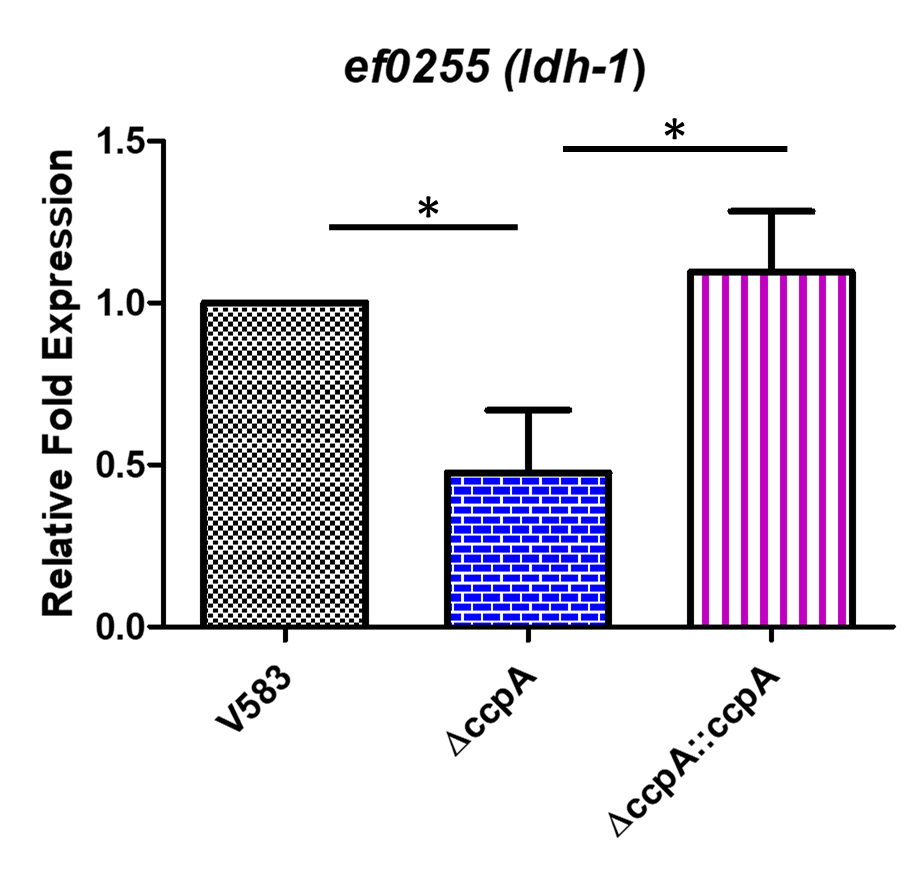
Figure S4**

qRT-PCR analysis of CcpA-dependent expression of *ef0255* (*ldh-1*). RNA was isolated from cultures of V583, Δ*ccpA*, and Δ*ccpA*::*ccpA* grown in CDM supplemented with 15mM glucose and subsequently converted to cDNA. The cDNA was subjected to qPCR analysis and quantified using the ΔΔCt method using the threshold cycle values for *ef0255* normalized to the endogenous control [*ef0005* (*gyrB*)]. Results represent averages of three independent biological experiments. Error bars indicate the standard deviation of the mean. Statistical analysis was done by one-way ANOVA, with significant values set to P < 0.05 (*).
