# Supplementary material for: Influence of the Alternative Sigma Factor RpoN on Global Gene Expression and Carbon Catabolism in Enterococcus faecalis V583
Source: mBio. 2021 May 18;12(3):e00380-21. doi: 10.1128/mBio.00380-21 (PMC8262876; doi:10.1128/mBio.00380-21)
Supplement: TABLE S1 [file mbio.00380-21-st001.docx]

**Table S1. *E. faecalis* strains used in this study**

| **Strain** | **Genotype or Description** | **Reference** |
| --- | --- | --- |
| V583 | Parental strain | Clinical isolate ([71](#_ENREF_71)) |
| VI01 | V583Δ*rpoN* | ([6](#_ENREF_6)) |
| VI40 | V583Δ*rpoN*::*rpoN* | ([6](#_ENREF_6)) |
| MG07 | V583Δ*mptR* | This study |
| MG07R | V583Ω*mptR* | This study |
| MG08 | V583Δ*lpoR* | This study |
| MG08R | V583Ω*lpoR* | This study |
| MG09 | V583Δ*mpoR* | This study |
| MG10 | V583Δ*mphR* | This study |
| IH10 | V583Δ*mptBACD* | This study |
| KC01 | V583Δ*xpoABCD* | This study |
| EH01 | V583∆*ccpA* | This study |
| EK26 | V583∆*ccpA*::*ccpA* | This study |
| ZA06 | V583Δ*ef2223-21* | This study |
| ZA10 | V583Δ*rpoN*Δ*ef2223-21* | This study |
| EK02 | V583Δ*ef1516* | This study |
