# Supplementary material for: Influence of the Alternative Sigma Factor RpoN on Global Gene Expression and Carbon Catabolism in Enterococcus faecalis V583
Source: mBio. 2021 May 18;12(3):e00380-21. doi: 10.1128/mBio.00380-21 (PMC8262876; doi:10.1128/mBio.00380-21)
Supplement: TABLE S2 [file mbio.00380-21-st002.docx]

Table S2. Oligonucleotides used in this study

| **Primer** | **Sequence (5'-3')** |
| --- | --- |
| LpoRP1 | GAGAGAATTCGATTGACAAGTTAAAAAAACG |
| LpoRP2 | CTCTGGATCCCTTCCGAGATTTCATGGAATC |
| LpoRP3 | GAGAGGATCCGAAGCGGAAGTTGCGCAT |
| LpoRP4 | CTCTCTGCAGGATAGCCAATGTACCATTCC |
| LpoR-Up | ATGCAAATTGCTGAAGTTGCT |
| LpoR-Down | GCTAGCTCATTCACATTGTAT |
| MphRP1 | GAGAGGATCCGCTTCAATATCATCAATCGTTTA |
| MphRP2 | CTCTTCTAGATAACATAAAATCACCTCTCGC |
| MphRP3 | GAGATCTAGAACGGAGTTTAACAACGCAGA |
| MphRP4 | CTCTCCATGGATGCAACCTAAAAGAATCGCT |
| MphRUp | GTGATGAAGTGAAATGCTTTC |
| MphRDown | CTTGAAAGCAACGAGGTTCC |
| MpoRP1 | GAGAGGATCCGAGCACATTCGTCAACGAATG |
| MpoRP2 | CTCTCTGCAGGTGAAACTGTTTTAACGCTTGATG |
| MpoRP3 | GAGACTGCAGAAGGCTTAATTCAAACGGAAATG |
| MpoRP4 | GCTGAGACAGCATGCCTGAA |
| MpoRUp | GCAGCAGTAACGATGGAAGA |
| MpoRDown | GTGGAGGTGACTCCGAGATA |
| MptRP1 | GAGAGAATTCATTTTGTCGCTAGTTGGCTTG |
| MptRP2 | CTCTGGATCCAATCCGCTCATTTTTGATGG |
| MptRP3 | GAGAGGATCCACCGAAATTGATACACTAGAT |
| MptRP4 | CTCTCTGCAGAAAGATCATTGCACCAGATTG |
| MptRUp | CGAGAGGAAGGCTTGAATGTC |
| MptRDown | CGATGCAATGGCTTCTTGCA |
| XpoABCDP1 | CAAGTGCTTCATGCTGGTGT |
| XpoABCDP2 | CTAAGTTTCCGTGACTAGC |
| XpoABCDP3 | CTAGGATTACTAGGCATCTGT |
| XpoABCDP4 | CTTCGTCCACGTTGATGTC |
| XpoABCDUp | CAAGTGATTGACTCCTTGGTT |
| XpoABCDDown | TTATCGACTTCCGTGACTAG |
| MptBACDP1 | GAAGACTATGAAATTATTGCG |
| MptBACDP2 | TAATCGATCATCAATTCGAGCTA |
| MptBACDP3 | GGCTTATTATAAGAATGACGAGG |
| MptBACDP4 | CAGTCCATTCCATGATGTTGT |
| MptBACDUp | TAGACTGGCATTTAGAAGTGA |
| MptBACDDown | GCAATTCTTTCATACGATTACG |
| CcpAP1 | GAGAGAATTCCAGAAGGTTCCAAGTAGCTG |
| CcpAP2 | CTCTGGATCCATTTGCCTCTCTAGCAACATC |
| CcpAP3 | GAGAGGATCCACAGTTGTTTTACCTTATGGAATTG |
| CcpAP4 | CTCTCTGCAGGACTTATGCTGATGGTCGTG |
| CcpA-Up | GCTGTAACACCAGGTTTCAC |
| CcpA-Down | GATCGTCAAGTTGGTTCTACG |
| EF0019f | ATTGGCGGAATGAGTTTCAG |
| EF0019r | GCTGGCGTTATTTTCTTTGC |
| EF2223f | AACATCGGCGGTATCTTCAG |
| EF2223r | TGGTTCAATTCGACGAACAA |
| EF0891f | TGTCACAGCAAGCAGGAATC |
| EF0891r | TTCCACAAAAGGAACGGAAG |
| EF0013f | TTGGATGATGCAGAACGAAG |
| EF0013r | CTTGCAAGCCAGCAGTCAT |
| EF0005f | GGTGTTGGTTCCTCTGTTGT |
| EF0005r | CGCACCACGACGATATTCTT |
| EF2223-21P1 | GAGAGGATCCTAACAAGTGTCGGTGATGAAC |
| EF2223-21P2 | CTCTTCTAGACTTCTTCATTCGTTTCGCTCCTTTC |
| EF2223-21P3 | GAGATCTAGAGAAATGCAAACACAATTAGACG |
| EF2223-21P4 | CTCTGCATGCCTTCTAAGTCAGGAAAGGTGC |
| EF2223-21Up | CGAAAATGGTGTCCTAAACAAAG |
| EF2223-21Down | CTTCTAAGTCAGGAAAGGTGC |
| EF0255f | CGGAGATACAGAATTCCCAGTTT |
| EF0255r | GCTTTAGTGATACGCGCTAGT |
| EF3210f | CTGAGGACTACACGCCAATTAT |
| EF3210r | TGACACTACCACCATATAAATCTGT |
| EF1017f | CGTGGTATGGAAGCAGACAT |
| EF1017r | ACGAACTTGTGGGCCTAATAA |
| EF1516P1 | GAGAGAATTCCAATGTGGTGTACAATGGTTAC |
| EF1516P2 | CTCTTCTAGACGCTTTCATTATACGTAACTCCT |
| EF1516P3 | GAGATCTAGAGATGTAATCGGAAATGTTACC |
| EF1516P4 | CTCTGCATGCATCAGTTACATCCCTTCTAGT |
| EF1516Up | CGAGCACATATAAGTATGCCT |
| EF1516Down | GAAGGCCTAACTCTTTCTGCTT |

Underlined sequences depict restriction sites used for cloning purposes
